# Patterns of Cough Medication Prescribing among Patients with Chronic Cough in Florida: 2012–2021

**DOI:** 10.3390/jcm12196286

**Published:** 2023-09-29

**Authors:** Seonkyeong Yang, Shu Huang, Juan M. Hincapie-Castillo, Xuehua Ke, Helen Ding, Jonathan Schelfhout, Mandel R. Sher, Bobby Jones, Debbie L. Wilson, Wei-Hsuan Lo-Ciganic

**Affiliations:** 1 Department of Pharmaceutical Outcomes & Policy, College of Pharmacy, University of Florida, Gainesville, FL 32611, USA; yang.se@ufl.edu (S.Y.); shu.huang@ufl.edu (S.H.); bobby.jones@ufl.edu (B.J.); debbie.wilson@ufl.edu (D.L.W.); 2 Department of Epidemiology, Gillings School of Global Public Health, University of North Carolina at Chapel Hill, Chapel Hill, NC 27599, USA; jhincapie-castillo@unc.edu; 3 Center for Observational and Real-World Evidence (CORE), Merck & Co., Inc., Rahway, NJ 07065, USA; xuehua.ke@merck.com (X.K.); helen.ding@merck.com (H.D.); jonathan.schelfhout@gmail.com (J.S.); 4 Center for Cough, Largo, FL 33778, USA; drmrsher@gmail.com; 5 Center for Drug Evaluation and Safety (CoDES), College of Pharmacy, University of Florida, Gainesville, FL 32610, USA

**Keywords:** cough, chronic cough, cough hypersensitivity syndrome, antitussive, opioid antitussive, benzonatate, dextromethorphan, gabapentinoid, group-based trajectory modeling, drug utilization

## Abstract

Among patients with chronic cough (CC) in the 2012–2021 statewide OneFlorida Clinical Research Consortium database, we examined trends in cough medication (CM) prescribing prevalence over time in repeated cross-sectional analyses and identified distinct CM utilization trajectories using group-based trajectory modeling (GBTM) in a retrospective cohort study. Among eligible adults (≥18 years) without cancer/benign respiratory tumor diagnoses, we identified CC patients and non-CC patients with any cough-related diagnosis. In the GBTM analysis, we calculated the number of monthly prescriptions for any CMs (excluding gabapentinoids) during the 12 months from the first qualifying cough event to identify distinct utilization trajectories. From 2012 to 2021, benzonatate (9.6% to 26.1%), dextromethorphan (5.2% to 8.6%), and gabapentinoid (5.3% to 14.4%) use increased among CC patients, while opioid antitussive use increased from 2012 to 2015 and decreased thereafter (8.4% in 2012, 14.7% in 2015, 6.7% in 2021; all *p* < 0.001). Of 15,566 CC patients and 655,250 non-CC patients identified in the GBTM analysis, CC patients had substantial burdens of respiratory/non-respiratory comorbidities and healthcare service and concomitant medication use compared to non-CC patients. Among CC patients, GBTM identified three distinct CM utilization trajectories: (1) no CM use (n = 11,222; 72.1%); (2) declining CM use (n = 4105; 26.4%); and (3) chronic CM use (n = 239; 1.5%). CC patients in Florida had limited CM use with increasing trends in use of benzonatate, dextromethorphan, and gabapentinoids and a decreasing trend in opioid antitussive use. CC patients, particularly with chronic prescription CM use, experienced substantial disease burden.

## 1. Introduction

Chronic cough (CC) is a cough persisting more than 8 weeks [1]. CC can be a severely debilitating condition with physical and mental health consequences [2,3,4]. The global prevalence of CC in adults was estimated to be approximately 10% in a meta-analysis, including varying definitions of CC from 90 studies [5]. In the United States (US), CC prevalence was reported to be 5% in a nationally representative survey sample of the general adult population with higher prevalence noted among women and older individuals [6,7]. Various respiratory/non-respiratory conditions and/or other factors (e.g., smoking, odors, airborne) can trigger or cause CC [8,9,10]. The most common causes of CC in patients with normal chest roentgenogram findings who are non-smokers and are not receiving therapy with angiotensin-converting enzyme (ACE) inhibitors are asthma, gastroesophageal reflux disease (GERD), upper airway cough syndrome (UACS)/rhinosinus conditions, and non-asthmatic eosinophilic bronchitis (NAEB) [1,8]. 

Clinical management of CC primarily focuses on the systematic assessment and treatment of underlying causes [11,12]. However, CC becomes refractory despite extensive diagnostic investigation and under guideline-based management for known causes or persists in some patients for unexplained reasons (hereafter referred to as refractory or unexplained CC [RCC/UCC]). Some studies have reported that patients with RCC/UCC universally present with an abnormally sensitive cough reflex [13,14]. Thus, emerging evidence suggests that RCC/UCC may be a single disease entity with neural dysregulation rather than a consequence of other underlying chronic conditions. A concept called cough hypersensitivity syndrome has been supported as a clinically useful paradigm to identify patients with RCC/UCC, characterized by troublesome coughing often triggered by low levels of thermal, mechanical, or chemical exposure [15]. Results from some randomized controlled trials (RCTs) suggest neuromodulators targeting hyperresponsiveness in the central nervous system (e.g., morphine, gabapentin, pregabalin, amitriptyline) alone or with speech pathology therapy can be a treatment option for RCC/UCC, although none of them have indication [16,17,18,19]. In 2016, the American College of Chest Physicians (CHEST) guideline recommended a trial of gabapentin for adults with RCC/UCC, as long as risk-benefit profiles of gabapentin therapy are carefully evaluated at initiation and reassessed at 6 months remaining on the use of the drug [20]. In 2020, the European Respiratory Society (ERS) guideline recommended a trial of low-dose (5−10 mg twice daily) morphine, gabapentin, or pregabalin in adults with RCC/UCC [15]. Although central-acting cough medications (CMs) (e.g., codeine, dextromethorphan) are recommended for chronic cough induced by certain underlying conditions (e.g., bronchitis, cancer), their efficacy for RCC/UCC has not been established in RCTs [1]. Since there are no therapies approved to treat RCC/UCC and few off-label therapies with some efficacy and safety data to treat the condition, commonly used CMs (e.g., codeine, dextromethorphan, benzonatate) may be taken by patients with RCC/UCC for symptomatic relief of cough, despite the lack of evidence on effectiveness.

There is limited literature on CM utilization patterns in patients with CC in real-world clinical settings. In this study, we aimed to characterize CC patients, examine the trends in CM utilization over time, and identify distinct CM utilization trajectories and associated factors in patients with CC in Florida using real-world healthcare data. 

## 2. Materials and Methods

### 2.1. Data Source

We used limited research data between 1 January 2012 and 31 December 2021 from the OneFlorida Data Trust (hereafter OneFlorida) that is established and managed by the OneFlorida Clinical Research Consortium. OneFlorida partners encompass academic healthcare centers, private and public health systems, community health centers, and physician-owned practices that provide care for ~50% of Floridians with diverse characteristics [21]. OneFlorida contains patient demographics and robust longitudinal clinical data, including electronic health records (EHR) and death data on >17 million unique individuals [22]. OneFlorida is currently one of the nation’s 9 clinical data research networks (CDRN), funded by the Patient-Centered Outcome Research Institute (PCORI) [22,23]. This study was approved by the University of Florida Institutional Review Board.

### 2.2. Study Design and Population

First, we conducted repeated cross-sectional analyses to estimate the annual CM prescribing prevalence and examine the trends in annual CM prescribing prevalence over time by 2 separate denominators: (1) CC patients; and (2) non-CC patients with any cough-related diagnosis. We excluded patients who were (1) aged < 18 years (measured on 30 June of each calendar year), (2) had any malignant cancer diagnoses or any respiratory tumor diagnoses, and (3) had <2 medical encounters in each calendar year. We used an existing algorithm that was used in previous studies to identify patients with CC based on the presence of any 3 clinical cough events occurring within a 120-day period and were separated from each other by at least 21 days (Figure S1) [24,25,26,27]. These cough events included a diagnosis of cough [ICD-9-CM: 786.2 or ICD-10-CM: R05] or a CM prescription order (opioid antitussives, benzonatate, or dextromethorphan-containing products). The first and the third events were required to be at least 56 days apart to meet the definition of CC (lasting ≥ 8 weeks). A validation study found this algorithm to have a low sensitivity (15.5%) but near-perfect specificity (99.6%) [24]. Although gabapentinoids (i.e., gabapentin and pregabalin) can be used for RCC/UCC based on the 2016 CHEST and 2020 ERS guidelines [15,20], we did not include gabapentinoids to identify cough events in the CC identification algorithm due to their largely off-label use for pain and other conditions [28]. Our second denominator (non-CC patients with any cough-related diagnosis) included individuals who were likely to have an acute or sub-acute cough (i.e., acute upper respiratory infections, influenza, bronchitis, pneumonia, cough, chronic upper respiratory tract diseases; list of diagnoses appear in Table S1).

Second, we conducted a retrospective cohort study using group-based trajectory modeling (GBTM) to identify distinct CM utilization trajectories over 12 months for CC patients and non-CC patients with any cough-related diagnosis, separately. We used the same operationalized definition of CC patients and non-CC patients with any cough-related diagnosis from the cross-sectional analyses. If more than 3 clinical cough events were found during the study period, the first 3 qualifying cough events were used to identify CC and determine an index date. The index date was defined as the date of a patient’s first qualifying cough event in the CC identification algorithm for CC patients (Figure S2) and the date of the first cough-related diagnosis for non-CC patients with any cough-related diagnosis (Figure S3). For the GBTM analysis, we excluded patients who were (1) aged < 18 years (measured on the index date), (2) had any malignant cancer diagnoses or any respiratory tumor diagnosis during the study period, (3) had an index date before 1 July 2012, or after 1 January 2021, and (4) had no encounter in the 6 months before the index date or had <2 encounters in the 12 months after the index date.

### 2.3. Outcomes of Interest

For the repeated cross-sectional analyses, the primary outcome was CM utilization patterns over time. CM classes of interest included (1) opioid antitussives (i.e., codeine monotherapy or codeine-, dihydrocodeine-, and hydrocodone-containing medications with antihistamines, nasal decongestants, or expectorants); (2) benzonatate; (3) dextromethorphan-containing medications with or without antihistamines, nasal decongestants, or expectorants); and (4) gabapentinoids (i.e., gabapentin and pregabalin). We used opioid analgesics (i.e., non-injectable opioid analgesics that are not used for opioid use disorder treatment) as a comparison group to provide trends in overall opioid use likely to be immensely affected by opioid policies/guidelines in the US [29,30]. All medications were identified in a prescribing dataset using RXCUI codes [31]. 

For the GBTM analysis, the primary outcome was the patient’s membership in a distinct trajectory of any CM use (i.e., opioid antitussives, benzonatate, and dextromethorphan-containing medications), excluding gabapentinoids. We identified these CM utilization trajectories by constructing monthly numbers of total prescriptions for any CM use in the 12 months after the index date. We did not include gabapentinoid use to identify trajectories due to their documented widespread off-label use for pain and other conditions [28]. 

### 2.4. Covariates

In the repeated cross-sectional analyses, we measured socio-demographic characteristics, including age (on 30 June of each calendar year), sex, race (i.e., White, Black, and others), major insurance type (i.e., Medicare, Medicaid, commercial, and others), and whether a patient resided in a metropolitan county.

In the GBTM analysis, we measured a series of patient-level covariates during the pre-index period (6 months before the index date) and post-index period (12 months after the index date). Socio-demographic characteristics included age (on the index date), sex, race (i.e., White, Black, and others), ethnicity (i.e., Hispanic and non-Hispanic/unknown), major insurance type (i.e., Medicare, Medicaid, commercial, and others), and whether a patient resided in a metropolitan county. Comorbidities included respiratory conditions (e.g., asthma, bronchitis, rhinosinus conditions), non-respiratory conditions (e.g., anxiety disorder, GERD, musculoskeletal disorders), and the modified Elixhauser Comorbidity Index (excluding metastatic cancers, solid tumors with or without metastasis, and diseases/conditions examined individually to avoid collinearity issues) [25,27,30,32]. Healthcare service use included categorical variables for any hospitalization, emergency department (ED) visit counts (0, 1, and ≥2), and outpatient visit counts (0, 1, 2–5, and ≥5), as well as a record of receiving medical procedures (e.g., bronchoscopy, chest X-ray, spirometry) [25,27]. We also examined various medications used in the pre-index period such as respiratory medications (e.g., asthma medications, antibiotics for respiratory conditions defined in Healthcare Effectiveness Data and Information Set [HEDIS] measures by the National Committee for Quality Assurance [NCQA] [33]), antidepressants, proton pump inhibitors, opioid analgesics, and polypharmacy with ≥3 different medications otherwise not ascertained individually in Tables S3–S6. 

During the post-index period in the GBTM analysis, we further examined additional covariates as follows: (1) the number of visits with a cough-related diagnosis; (2) the number of CM orders excluding gabapentinoids and the number of CM orders including gabapentinoids; (3) presence of specialist visits; and (4) encounter-level prescriber specialty for any CM orders excluding gabapentinoids.

### 2.5. Statistical Analysis

In the repeated cross-sectional analyses, the annual prevalence of prescriptions for the 5 medication groups (i.e., opioid antitussives, benzonatate, dextromethorphan-containing medications, gabapentinoids, and opioid analgesics) were estimated among CC patients and non-CC patients with any cough-related diagnosis that was separate in each calendar year from 2012 to 2021. Next, we tested the significance of trends in the annual prescribing prevalence over time using multivariable generalized estimating equations adjusted for age, sex, race, major insurance type, and residence in metropolitan counties. 

In the GBTM analysis, we built models using the longitudinal count of the number of monthly prescriptions for any CM (excluding gabapentinoids) as the outcome variable and the month since the index date (1−12 month) was the time variable. In the GBTM models, we used zero-inflated Poisson distribution with the most flexible functional form of time (e.g., up to the fifth-order polynomial function of time) to allow the trajectories to emerge from the data. GBTM outputs included estimated probabilities of group membership for each individual, estimated trajectory curves over time, and the proportion of membership in each group trajectory. The final GBTM models were selected based on a combination of (1) the Bayesian information criterion (BIC, Schwarz formulation), wherein the largest value indicates the best-fitting model, (2) application of Nagin’s criteria to assess final model adequacy [34,35,36], and (3), as conducted in prior studies, clinically interpretable trajectories with a minimum proportion of the cohort (e.g., 5%) for each trajectory that might result in effective future interventions based on our discussion with the clinical expert on the team [37,38]. Nagin’s criteria for a well-performing trajectory model include an average posterior probability of ≥0.7 for all groups, an odds of correct classification of ≥5.0 for all groups, and narrow confidence intervals for estimated group membership probabilities [36]. GBTMs were estimated using traj in STATA 17 (StataCorp LLC, College stations, TX, USA). 

We presented descriptive data for pre-index and post-index characteristics of CC patients and non-CC patients with any cough-related diagnosis. We compared those characteristics between CC patients and non-CC patients with any cough-related diagnosis using the chi-square test, Fisher’s exact test, or Student’s t-test, as appropriate. In addition, we compared characteristics across the CM utilization trajectories identified in CC patients and non-CC patients with any cough-related diagnosis, separately, using the chi-square test, Fisher’s exact test, or Analysis of Variance, as appropriate. We also examined the association between pre-index factors and membership in a trajectory group using multinomial logistic regression modeling in CC patients. A stepwise variable selection approach (significance level for entry = 0.05; for stay = 0.01) was used to identify pre-index factors associated with CM utilization trajectories. Multicollinearity between pre-index factors was assessed using variance inflation factor. Finally, we performed multinomial logistic regression models, including the selected characteristics from the previous steps. We reported adjusted odds ratios (ORs) with a 95% confidence interval (CI). Statistical significance was defined as *p* < 0.05 (2-tailed). All analyses, except GBTM, were conducted in SAS version 9.4 (SAS Institute Inc., Cary, NC, USA).

### 2.6. Secondary Analyses

In the secondary analyses, we included gabapentinoids in the GBTM analysis and explored the trajectories across drug classes to provide a context for our primary GBTM analysis for any CM use excluding gabapentinoids. We identified CM utilization trajectories by constructing monthly numbers of total prescriptions for each CM class (i.e., opioid antitussives, benzonatate, dextromethorphan-containing medications, and gabapentinoids) and any CM use including gabapentinoids in the 12 months after the index date.

## 3. Results

### 3.1. Repeated Cross-Sectional Analyses: 2012–2021 OneFlorida Data

#### 3.1.1. Trends in Annual CM Prescribing Prevalence in CC Patients 

**Figure 1:**
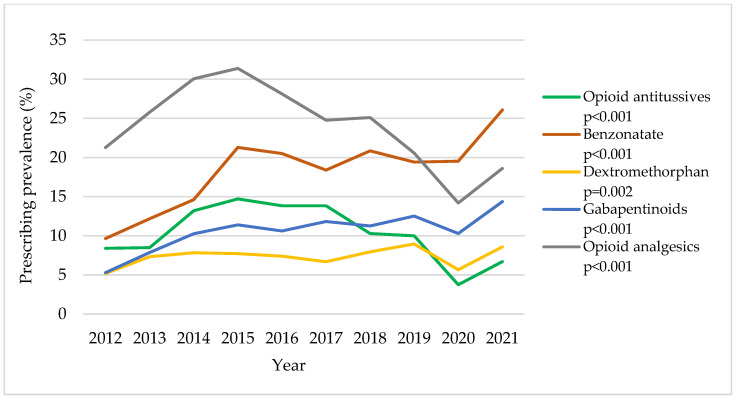
Trends in annual cough medication (CM) prescribing prevalence in patients with chronic cough (CC): 2012–2021 OneFlorida data. All *p*-values were adjusted for age, sex, race, major insurance type, and residence in metropolitan counties in multivariable generalized estimating equations. The denominator (CC patients) is 964; 1462; 1697; 1931; 2233; 2572; 2605; 2324; 2720; and 1956 for each calendar year, respectively, in ascending order.

Among CC patients (Table S2 and Figure 1), there were significant increasing trends in benzonatate use (from 9.6% in 2012 to 26.1% in 2021; adjusted *p* < 0.001), dextromethorphan use (from 5.2% in 2012 to 8.6% in 2021; adjusted *p* = 0.002), and gabapentinoid use (from 5.3% to 14.4%; adjusted *p* < 0.001). Opioid antitussive use increased until 2015 and then decreased thereafter (8.4% in 2012, 14.7% in 2015, and 6.7% in 2021; adjusted *p* < 0.001). The trend in opioid analgesic use showed a similar trend as opioid antitussives but with a greater overall prescribing prevalence (21.3% in 2012, 31.4% in 2015, and 18.6% in 2021; adjusted *p* < 0.001). Overall, our analysis reveals increasing trends in the prescribing prevalence of medications of interest between 2020 and 2021. 

#### 3.1.2. Trends in Annual CM Prescribing Prevalence in Non-CC Patients with Any Cough-Related Diagnosis

**Figure 2:**
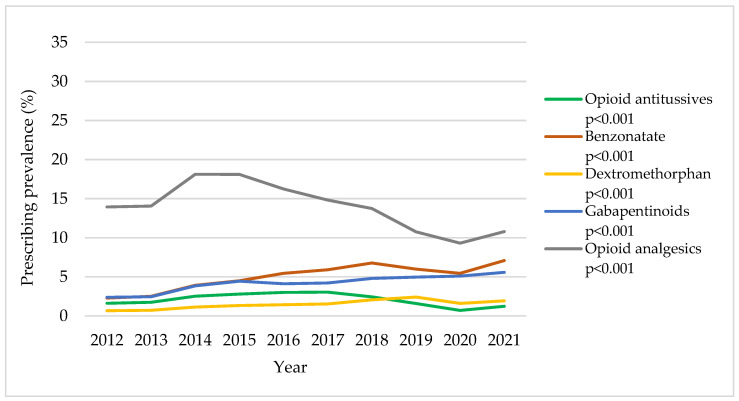
Trends in annual cough medication (CM) prescribing prevalence in non-chronic cough (CC) patients with any cough-related diagnosis: 2012–2021 OneFlorida data. All *p*-values were adjusted for age, sex, race, major insurance type, and residence in metropolitan counties in multivariable generalized estimating equations. The denominator (non-CC patients with any cough-related diagnosis) is 179,637; 222,940; 236,606; 242,860; 284,538; 300,353; 297,809; 284,174; 299,467; and 317,434 for each calendar year in ascending order.

While the trends in annual CM prescribing prevalence among non-CC patients with any cough-related diagnosis are consistent with the trends among CC patients, there was an overall lower prescribing prevalence across study years for the population of non-CC patients. Among non-CC patients with any cough-related diagnosis (Table S2 and Figure 2), there were significant increasing trends in benzonatate use (from 2.3% in 2012 to 7.1% in 2021), dextromethorphan use (from 0.7% in 2012 to 1.9% in 2021), and gabapentinoid use (from 2.4% to 5.6%). Opioid antitussive use increased until 2017, and then decreased thereafter (1.6% in 2012, 3.0% in 2017, and 1.2% in 2021; all adjusted *p* < 0.001). Opioid analgesic use in non-CC patients with any cough-related diagnosis had a similar trend as in CC patients but with a lower prescribing prevalence (13.9% in 2012, 18.1% in 2014, and 10.8% in 2021; adjusted *p* < 0.001). Overall, we observed increasing trends in the prescribing prevalence of medications of interest between 2020 and 2021.

### 3.2. GBTM Analysis: 2012–2021 OneFlorida Data

#### 3.2.1. Distinct CM Utilization Trajectories Identified in CC Patients and Non-CC Patients with Any Cough-Related Diagnosis

From a total of 16,697,875 individuals in the 2012−2021 OneFlorida data, we identified 15,566 CC patients and 655,260 non-CC patients with any cough-related diagnosis who met inclusion and exclusion criteria for the retrospective cohort study (Figure S4).

**Figure 3:**
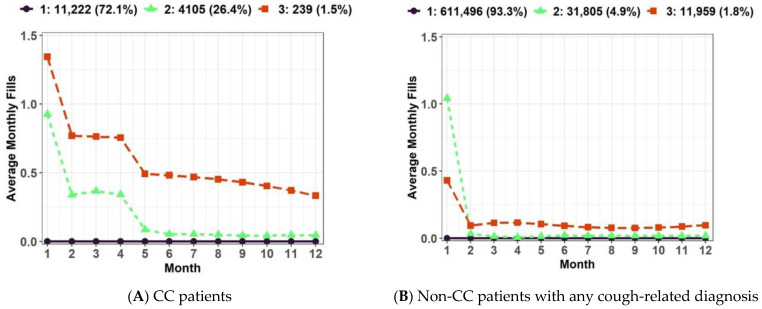
Trajectories of cough medication (CM) utilization excluding gabapentinoids: (**A**) Three distinct trajectories were identified in CC patients; (**B**) Three distinct trajectories were identified in non-CC patients with any cough-related diagnosis. Abbreviation: CC: Chronic Cough.

We identified three distinct CM utilization trajectories in CC patients (Figure 3); (1) Group 1: no CM use (n = 11,222; 72.1%), (2) Group 2: declining CM use (n = 4105; 26.4%), and (3) Group 3: chronic CM use (n = 239; 1.5%). We identified three distinct CM utilization trajectories in non-CC patients with any cough-related diagnosis (Figure 3); (1) Group 1: no CM use (n = 611,496; 93.3%), (2) Group 2: declining CM use (n = 31,805; 4.9%), and (3) Group 3: chronic minimal CM use (n = 11,959; 1.8%). 

#### 3.2.2. Pre-Index Characteristics of CC Patients and Non-CC Patients with Any Cough-Related Diagnosis

**Figure 4:**
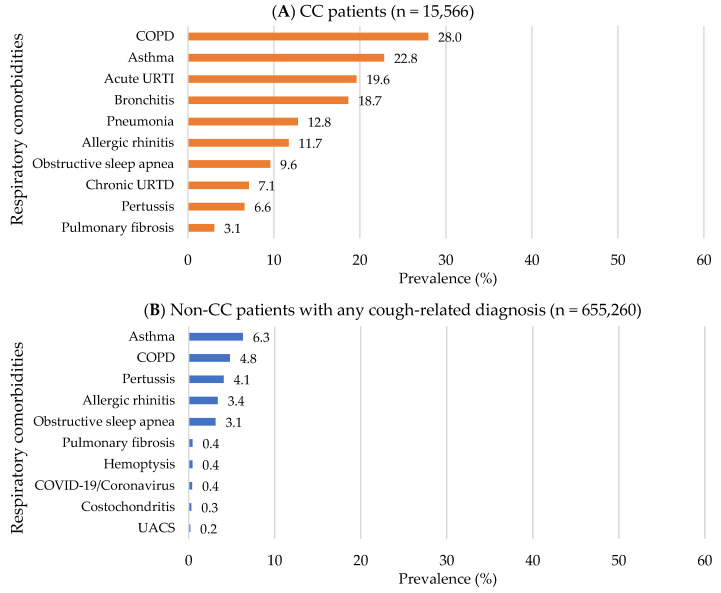
Top 10 pre-index respiratory comorbidities: 2012−2021 OneFlorida data. (**A**) In CC patients; (**B**) In non-CC patients with any cough-related diagnosis. Abbreviations: CC: Chronic Cough; COPD: Chronic Obstructive Pulmonary Disease; URTI: Upper Respiratory Tract Infection; URTD: Upper Respiratory Tract Disease; COVID-19: Coronavirus Disease caused by the SARS-CoV-2 virus; UACS: Upper Airway Cough Syndrome.

**Figure 5:**
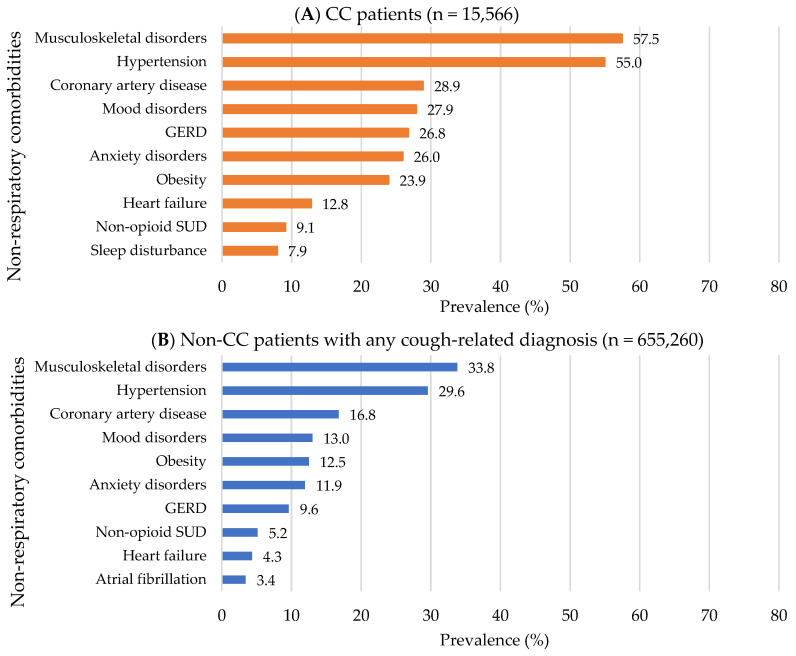
Top 10 pre-index non-respiratory comorbidities: 2012−2021 OneFlorida data. (**A**) In CC patients; (**B**) In non-CC patients with any cough-related diagnosis. Abbreviations: CC: Chronic Cough; GERD: Gastroesophageal Reflux Disease; SUD: Substance Use Disorder.

**Figure 6:**
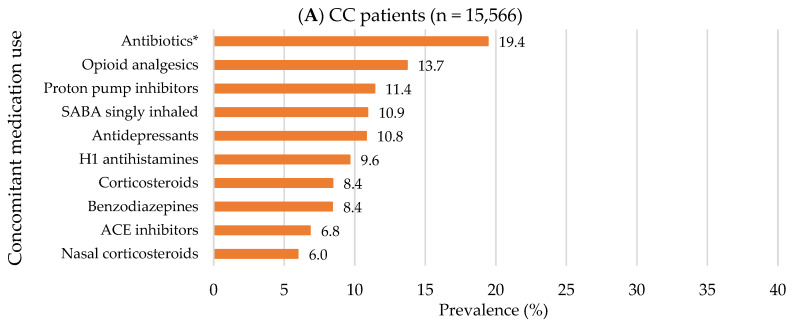

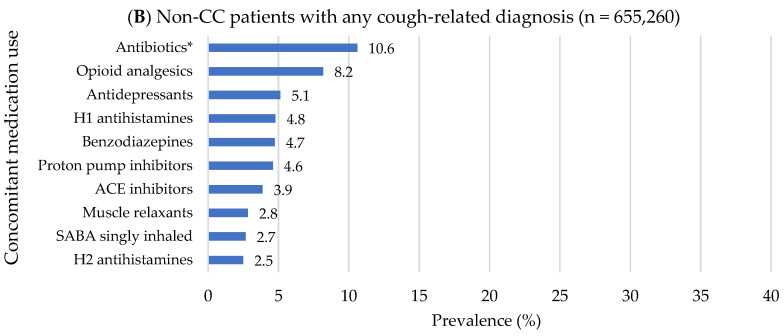
Top 10 pre-index concomitant medication use: 2012−2021 OneFlorida data. (**A**) In CC patients; (**B**) In non-CC patients with any cough-related diagnosis. Abbreviations: CC: Chronic Cough; SABA: Short-acting Beta-agonist; H1: Histamine-1 receptor; ACE: Angiotensin Converting Enzyme; H2: Histamine-2 receptor. * Antibiotics for respiratory conditions.

Compared to non-CC patients with any cough-related diagnosis, CC patients were more likely to be older (mean age for CC vs. non-CC patients: 53.9 ± 18.2 vs. 46.3 ± 19.4 years), female (70.2% vs. 68.6%) and to have Medicare as a major payer (48.1% vs. 31.1; all *p* < 0.001; Table S3). In addition, CC patients had approximately two-fold higher healthcare service use: ≥1 hospitalization (25.7% vs. 13.5), ≥2 ED visits (33.0% vs. 17.4%), and ≥5 outpatient visits (64.2% vs. 35.2%; all *p* < 0.001; Table S3). The top three most common pre-index respiratory comorbidities in CC patients were chronic obstructive pulmonary disease (COPD) (28.0%), asthma (22.8%), and acute URTIs (19.6%), while the top three for non-CC patients were asthma (6.3%), COPD (4.8%), and pertussis (4.1%; Table S3 and Figure 4). The top three most common pre-index non-respiratory comorbidities were the same for both groups but with a higher prevalence in CC patients compared to non-CC patients as follows: musculoskeletal disorders (57.5% vs. 33.8%), hypertension (55.0% vs. 29.6%), and coronary artery disease (28.9% vs. 16.8%; all *p* < 0.001; Table S3 and Figure 5). In addition, CC patients had a higher Elixhauser comorbidity index than non-CC patients (1.7 ± 1.9 vs. 0.7 ± 1.3; *p* < 0.001; Table S3). Overall, concomitant medication use was prevalent in CC patients compared to non-CC patients (Table S3 and Figure 6). The top three most frequently used pre-index concomitant medications in CC patients were antibiotics for respiratory conditions (19.4%), opioid analgesics (13.7%), and proton pump inhibitors (11.4%), while the top three for non-CC patients were antibiotics for respiratory conditions (10.6%), opioid analgesics (8.2%), and antidepressants (5.1%). In addition, CC patients with chronic CM use had a substantial burden of respiratory/non-respiratory comorbidities, healthcare utilization, and concomitant medication use than other trajectory groups in the pre-index period (Table S4). Non-CC patients with any cough-related diagnosis having chronic minimal CM use had an increased concomitant medication use compared to other trajectory groups in the pre-index period (Table S5).

#### 3.2.3. Post-Index Characteristics of CC Patients and Non-CC Patients with Any Cough-Related Diagnosis

Similar to the pre-index characteristics, CC patients had a higher burden of post-index respiratory/non-respiratory comorbidities and concomitant medication use (Table S6 and Figures S5−S7). Post-index CM prescribing prevalence is as follows (in descending order in CC patients, and were significantly higher compared to non-CC patients with any cough-related diagnosis): benzonatate (19.7% vs. 4.4%), opioid antitussives (11.2% vs. 2.1%), gabapentinoids (10.3% vs. 3.9%), dextromethorphan (7.7% vs. 1.2%; all *p* < 0.001; Table S6 and Figure S8). Compared to non-CC patients with any cough-related diagnosis, CC patients were more likely to visit pulmonologist (37.7% vs. 7.1%), gastroenterologist (23.2% vs. 8.3%), and otolaryngologist (12.7% vs. 4.0%; all *p* < 0.001; Table S6). In both groups, more than half of CMs (i.e., opioid antitussive, benzonatate, and dextromethorphan) were prescribed by primary care providers (58.4% vs. 54.4%) and a very limited number of prescriptions came from cough specialists (i.e., allergists, otolaryngologists, pulmonologist) during the trajectory measurement period (Table S7). In addition, CC patients with chronic CM use had a substantial burden of respiratory/non-respiratory comorbidities, healthcare utilization, and concomitant medication use than other trajectory groups in the post-index period (Table S8). Non-CC patients with any cough-related diagnosis having chronic minimal CM use had a higher prevalence of respiratory comorbidities and concomitant medication use than other trajectory groups in the post-index period (Table S9).

#### 3.2.4. Pre-Index Factors Associated with CM Utilization Trajectories in CC Patients

Compared to Group 1 (no CM use), factors found to be significantly positively associated with both Group 2 (declining CM use) and Group 3 (chronic CM use) in CC patients were Black race, having commercial health insurance, asthma, pulmonary fibrosis, obesity, polypharmacy, and the previous use of H_1_ antihistamines, nasal/oral corticosteroids, potential respiratory antibiotics, muscle relaxants, and opioid analgesics (Table S10 and Figure S9). Compared to Group 1, factors found to be significantly negatively associated with both Groups 2 and 3 in CC patients were having Medicaid insurance, musculoskeletal disorder, and orders for complete blood cell count, chest X-ray, and spirometry (Table S10 and Figure S9).

### 3.3. Secondary Analyses 

CM utilization trajectories for each CM class (i.e., opioid antitussives, benzonatate, dextromethorphan-containing medications, and gabapentinoids) and any CM including gabapentinoids are presented in Figures S10−S14. We observed similar patterns in the three trajectories identified for opioid antitussive, benzonatate, and dextromethorphan-containing medication utilization in CC patients as CM (excluding gabapentinoids) utilization trajectories in primary analysis. The declining pattern in gabapentinoid use among CC patients was less apparent compared to other CM utilization trajectories.

## 4. Discussion

Using Florida statewide clinical data, our study yielded several important findings that help to better understand the clinical characteristics and CM use patterns of CC patients. The repeated cross-sectional analyses using Florida statewide clinical data showed statistically significant increasing trends in benzonatate, dextromethorphan, and gabapentinoid prescribing in CC patients from 2012 to 2021. In contrast, opioid antitussive prescribing increased from 2012 until 2015 and then decreased, which was similar to the trend in opioid analgesic prescribing over time. Benzonatate remained the most frequently prescribed CM among CC patients across all years. We observed similar trends among non-CC patients with any cough-related diagnosis with an overall lower CM prescribing prevalence. The observed substantial increase in benzonatate use in Florida is consistent with previous studies conducted in the US using nationally representative data [39,40]. 

To mitigate the US drug overdose crisis, numerous policies and guidelines have been formulated to avoid potentially harmful opioid prescribing in the past decade [29,30]. Those policies/guidelines mainly focus on opioid analgesic use for pain management, and do not provide specific recommendations for opioid antitussive use. However, the similar decreasing trends in both opioid antitussive and opioid analgesic prescribing suggest that opioid policies may have affected the decrease in opioid antitussive use in recent years. In addition, given an increasing trend in gabapentinoid use reported for a wide range of off-label indications in the general population [41,42,43,44,45], it is unlikely that the observed increasing trend in gabapentinoid prescribing among CC patients in our study is largely attributable to their use to treat RCC/UCC. It is important to note, though, that the prescribing prevalence of gabapentinoids was two- to three-fold higher in CC patients compared to non-CC patients with any cough-related diagnosis. Furthermore, overall increasing trends in all CM use from 2020 to 2021 were likely to be affected by the COVID-19 pandemic. One of common symptoms of COVID-19 infection is cough, which may require CM use [46]. As the main intent of this study was not to evaluate the changes in trends of CM use after the onset of the COVID-19 pandemic, we limit our discussion to overall observed trends over the entire study period (i.e., 2012 to 2021).

The GBTM analysis to identify distinct CM utilization trajectories yielded several important findings. First, most CC patients (~72%) were not prescribed any CMs during the 12-month trajectory measurement period. There could be several explanations for this group of CC patients. Since the clinical management of CC primarily focuses on the detection and treatment of underlying conditions [1], cough in non-CM users among CC patients might have been managed or resolved by successful treatments for underlying conditions without additional need for CM use. Another potential explanation could be that non-CM users among CC patients had less severe cough symptoms that might obtain over-the-counter CMs and did not necessitate CM prescription use. Second, we identified a group of CC patients with sustained CM use (average number of any CM prescriptions excluding gabapentinoids: 8.1 ± 3.0) during the 12-month trajectory period—this finding is likely to represent those patients with RCC/UCC. Sustained CM users among CC patients had a substantial burden of respiratory and non-respiratory comorbidities, healthcare utilization, and concomitant medication use in the pre-index and post-index periods. Third, the vast majority of non-CC patients with any cough-related diagnosis (~93%) were not prescribed any CMs, and CM prescriptions for the remaining non-CC patients were provided only during the first month after the cough-related diagnosis. Given that most cough-related diagnoses (e.g., acute URTI and bronchitis) are usually self-limited, no CM use or acute CM use seems reasonable among non-CC patients with any cough-related diagnosis. 

We observed that individuals with CC were found to have a substantial burden of respiratory and non-respiratory comorbidities, healthcare utilization, and concomitant medication use. The prevalence of common causes of CC (e.g., asthma, GERD, and UACS) was substantially higher in CC patients than in non-CC patients with any cough-related diagnosis during the pre-index and post-index periods. Furthermore, CC patients were more likely to be obese, have musculoskeletal disorders (e.g., arthritis and pain conditions), anxiety disorder, and mood disorder, and receive prescriptions for opioid analgesics compared to non-CC patients with any cough-related diagnosis, which is substantiated in the literature. For instance, Colak et al. identified obesity as a risk factor for chronic cough in a population-based survey data linked to the Danish National Patient Registry [47]. Also, emerging evidence suggests that chronic cough is a neuropathic condition with neuronal hypersensitivity on peripheral or central neural pathways mediating cough [13,14,48,49]. Thus, there might be a common pathophysiology shared between chronic cough and chronic pain. A recent study showed a bidirectional association between chronic cough and chronic pain, where chronic pain was identified as a risk factor for incident chronic cough and chronic cough was identified as a risk factor for incident chronic pain [50]. In addition, it has been reported that chronic cough is associated with depression [51,52] and impaired quality of life [3,48,53]. 

The higher healthcare service use in CC patients, including hospitalization and ED visits, may reflect the high burden of disease in CC patients compared to non-CC patients. In addition, we observed that 37.7% of CC patients had ≥1 pulmonologist visit, 23.2% had ≥1 gastroenterologist visit, 12.7% had ≥1 otolaryngologist visit, which were substantially higher than those without CC (7.1%, 8.3%, 4.0%, respectively). Management of CC requires an interdisciplinary approach involving primary and specialty care [54,55]. Primary care physicians (PCPs) can play an important role in providing the initial diagnostic assessment of CC patients as well as trials of empirical therapies for potential underlying causes. Specialty care is essential for advanced diagnostic testing for uncommon causes of CC and providing optimal care to patients with RCC/UCC. In our study, the majority of the CM prescriptions were prescribed by primary care physicians, and a very limited number of CM prescriptions came from cough specialists, which could indicate the following: (1) PCPs may primarily focus on immediate symptom relief in CC patients, while cough specialists are more likely to focus on treating underlying conditions and functional recovery; or (2) only a small subset of CC patients may belong to RCC/UCC patients who are likely to seek care from cough specialists. 

Finally, this study shows that although in CC patients there was an increase in CM use during the study period, the most common treatment patterns were the treatment of the underlying condition. The fact that ~72% of CC patients had no CM use, in addition to the fact that the percentage of chronic CM use was similar in CC and non-CC patients, means that other treatments may be the basis for the treatment of CC. Our finding in the post-index period showed that short-acting beta-agonist (SABA), H_1_ antihistamines, nasal corticosteroids, SABA/short-acting muscarinic-antagonist (SAMA), inhaled corticosteroid (ICS)/long-acting beta-agonist (LABA), and proton pump inhibitors are commonly used, and is in line with several publications that explore the most commonly used drugs in treatment for CC, which were inhaled bronchodilators, inhaled corticosteroids [56,57]; or topical corticosteroids, inhaled corticosteroids, inhaled bronchodilators, and proton pump inhibitors [58]. The reasons for this are diverse, with the lack of efficacy of the current CM to treat CC conflicting with the high need of these patients to find something to alleviate their CC. The new treatments that are under development that specifically target the mechanism of disease in RCC/UCC will probably change this treatment pattern in the future and, most importantly, will help to improve RCC/UCC patients’ lives. In addition, it is noteworthy to mention the high antibiotic use in CC patients that has also been shown in previous CC study [59]. Such use may reflect COPD exacerbations but also imply an abuse of antibiotics that may cause antibiotic resistance in the future.

This study has several limitations. First, there are several potential reasons for the underestimation of CC prevalence, as follows: (1) the ICD-9/10-CM code for cough may not capture all patients with cough. The ICD-9/10-CM code for cough is found under the signs and symptoms section (i.e., ICD-9-CM: 780−789; ICD-10-CM: R00−R69). Codes in this section are only used when signs or symptoms are not explained by the progress of an underlying disease. (2) Since the ICD-9/10-CM codes for cough within our study period do not provide information on the duration of cough, we may have misclassified some CC patients as non-CC patients when they encountered healthcare services after their cough persisted for more than 8 weeks and did not have subsequent follow-up visits. This issue might subside in the future given that since 1 October 2021, ICD-10-CM codes for specific cough based on the length of duration (i.e., R05.1: acute cough; R05.2: subacute cough; R05.3: chronic cough) are available. (3) We used the prescribing dataset in the OneFlorida data to identify written prescribing orders for CM; thus, there is lack of information on CM actually dispensed or refills. (4) We also could not capture over-the-counter dextromethorphan use. (5) There was a high percentage of missingness (~30%) in the provider specialty variable to determine rendering provider and prescribing provider. (6) In addition, our study was limited to using structured data fields in the OneFlorida data; thus, information documented in unstructured clinical data (e.g., duration of cough symptoms and severity) was not captured. Although previous studies demonstrated that the use of natural language processing (NLP) in unstructured data significantly improved sensitivity in identifying CC patients in EHR data [25,26], using an NLP-integrated algorithm to identify CC is not widely used because it can be time-consuming and computationally intensive in clinical practice. In sum, the CC prevalence in our analysis is likely to be substantially underestimated, although there is a possibility that we may have misclassified uncontrolled COPD patients as CC patients, based on the fact that CC patients had COPD as the most common pre-index respiratory comorbidity. Nevertheless, the CC identification algorithm we used has near-perfect specificity (99.6%) [24]; thus, it is still a clinically meaningful method for characterizing the CC patients identified in our study. Of note, the current analysis did not permit identification of those with RCC/UCC, so our results apply to the general CC population. Finally, it is important to consider that the OneFlorida data generalizes patients in the State of Florida; however, the findings might not necessarily be transferable to other states or US national estimates. Nonetheless, Florida is one of the largest and most diverse states in the US and the results can be informative for policy and clinical decision-making. 

## 5. Conclusions

In Florida, the prescribing prevalence of benzonatate, dextromethorphan, and gabapentinoid use increased among CC patients from 2012, while the prescribing prevalence of opioid antitussives increased until 2015 and then decreased by 2021. Individuals with CC were found to have a substantial burden of respiratory and non-respiratory comorbidities, healthcare utilization, and concomitant medication use. CC patients had low chronic antitussive use, which may indicate unmet or suboptimal management of CC in a subset of CC patients, potentially those with RCC/UCC. CC patients with chronic prescription CM use experienced substantial disease burden at baseline. Future studies are warranted to further explore underlying reasons for increased healthcare utilization in CC patients, identify patients with RCC/UCC, and characterize their treatment patterns. 

## Data Availability

RXCUI codes can be provided by request to the corresponding author. Modeling codes can be provided by request to the corresponding author under proper use agreement. Access to the OneFlorida data was made possible through an agreement between the University of Florida and the OneFlorida Clinical Research Consortium. Per the data use agreements, the relevant limited data sets utilized in this study contained some patient health information variables (e.g., dates of services) and thus cannot be made publicly available. This study was approved by the University of Florida Institutional Review Boards (IRB; human research ethics committees). Patient consent was waved for this study due to the use of existing secondary data sources per the IRB’s policies.

## Supplementary Materials

The following are available online at https://www.mdpi.com/article/10.3390/jcm12196286/s1, Table S1: ICD-9/10-CM codes to identify cough-related diagnosis, Table S2: Trends in annual cough medication (CM) prescribing prevalence in 2012–2021 OneFlorida data, Table S3: Pre-index characteristics of chronic cough (CC) patients and non-CC patients with any cough-related diagnosis, Table S4: Pre-index characteristics of chronic cough (CC) patients by cough medication (CM) utilization trajectory group, Table S5: Pre-index characteristics of non-chronic cough (CC) patients with any cough-related diagnosis by cough medication (CM) utilization trajectory group, Table S6: Post-index characteristics of chronic cough (CC) patients and non-CC patients with any cough-related diagnosis, Table S7: Prescribing specialty for all post-index cough medication (CM) prescription orders by CM utilization trajectory group, Table S8: Post-index characteristics of chronic cough (CC) patients by cough medication (CM) utilization trajectory group, Table S9: Post-index characteristics of non-chronic cough (CC) patients with any cough-related diagnosis by cough medication (CM) utilization trajectory group, Table S10: Odds ratios for pre-index factors associated with cough medication (CM) utilization trajectories in chronic cough (CC) patients, Figure S1: Chronic cough identification algorithm, Figure S2: Study design diagram for chronic cough (CC) patients in group-based trajectory modeling analysis, Figure S3: Study design diagram for non-chronic cough (CC) patients with any cough-related diagnosis in group-based trajectory modeling (GBTM) analysis, Figure S4: Flowchart for group-based trajectory modeling (GBTM) analysis: 2012−2021 OneFlorida data, Figure S5: Top 10 post-index respiratory comorbidities: 2012−2021 OneFlorida data, Figure S6: Top 10 post-index non-respiratory comorbidities: 2012−2021 OneFlorida data, Figure S7: Top 10 post-index concomitant medication use: 2012−2021 OneFlorida data, Figure S8: Post-index cough medication (CM) use: 2012−2021 OneFlorida data, Figure S9: Forest plot showing odds ratios for factors associated with cough medication (CM) utilization trajectories in chronic cough (CC) patients, Figure S10: Trajectories of opioid antitussive utilization: 2012−2021 OneFlorida data, Figure S11: Trajectories of benzonatate utilization: 2012−2021 OneFlorida data, Figure S12: Trajectories of dextromethorphan-containing medication utilization: 2012−2021 OneFlorida data, Figure S13: Trajectories of gabapentinoid utilization: 2012−2021 OneFlorida data, Figure S14: Trajectories of cough medication (CM) utilization including gabapentinoids: 2012−2021 OneFlorida data.

## References

[1] Irwin R.S., Baumann M.H., Bolser D.C., Boulet L.P., Braman S.S., Brightling C.E., Brown K.K., Canning B.J., Chang A.B., Dicpinigaitis P.V. (2006). Diagnosis and management of cough executive summary: ACCP evidence-based clinical practice guidelines. Chest.

[2] Koskela H.O., Latti A.M., Pekkanen J. (2018). The impacts of cough: A cross-sectional study in a Finnish adult employee population. ERJ Open Res..

[3] French C.L., Irwin R.S., Curley F.J., Krikorian C.J. (1998). Impact of chronic cough on quality of life. Arch. Intern. Med..

[4] Chamberlain S.A., Garrod R., Douiri A., Masefield S., Powell P., Bucher C., Pandyan A., Morice A.H., Birring S.S. (2015). The impact of chronic cough: A cross-sectional European survey. Lung.

[5] Song W.J., Chang Y.S., Faruqi S., Kim J.Y., Kang M.G., Kim S., Jo E.J., Kim M.H., Plevkova J., Park H.W. (2015). The global epidemiology of chronic cough in adults: A systematic review and meta-analysis. Eur. Respir. J..

[6] Meltzer E.O., Zeiger R.S., Dicpinigaitis P., Bernstein J.A., Oppenheimer J.J., Way N.A., Li V.W., Boggs R., Doane M.J., Urdaneta E. (2021). Prevalence and Burden of Chronic Cough in the United States. J. Allergy Clin. Immunol. Pract..

[7] McGarvey L., Rubin B.K., Ebihara S., Hegland K., Rivet A., Irwin R.S., Bolser D.C., Chang A.B., Gibson P.G., Mazzone S.B. (2021). Global Physiology and Pathophysiology of Cough: Part 2. Demographic and Clinical Considerations: CHEST Expert Panel Report. Chest.

[8] Pratter M.R. (2006). Overview of common causes of chronic cough: ACCP evidence-based clinical practice guidelines. Chest.

[9] Sandage M.J., Ostwalt E.S., Allison L.H., Cutchin G.M., Morton M.E., Odom S.C. (2021). Irritant-Induced Chronic Cough Triggers: A Scoping Review and Clinical Checklist. Am. J. Speech Lang. Pathol..

[10] Zhang J., Perret J.L., Chang A.B., Idrose N.S., Bui D.S., Lowe A.J., Abramson M.J., Walters E.H., Lodge C.J., Dharmage S.C. (2022). Risk factors for chronic cough in adults: A systematic review and meta-analysis. Respirology.

[11] Irwin R.S., French C.L., Chang A.B., Altman K.W., Panel C.E.C. (2018). Classification of Cough as a Symptom in Adults and Management Algorithms: CHEST Guideline and Expert Panel Report. Chest.

[12] Smith J.A., Woodcock A. (2016). Chronic Cough. N. Engl. J. Med..

[13] Vertigan A.E., Gibson P.G. (2011). Chronic refractory cough as a sensory neuropathy: Evidence from a reinterpretation of cough triggers. J. Voice.

[14] Song W.J., Kim J.Y., Jo E.J., Lee S.E., Kim M.H., Yang M.S., Kang H.R., Park H.W., Chang Y.S., Min K.U. (2014). Capsaicin cough sensitivity is related to the older female predominant feature in chronic cough patients. Allergy Asthma Immunol. Res..

[15] Morice A.H., Millqvist E., Belvisi M.G., Bieksiene K., Birring S.S., Chung K.F., Dal Negro R.W., Dicpinigaitis P., Kantar A., McGarvey L.P. (2014). Expert opinion on the cough hypersensitivity syndrome in respiratory medicine. Eur. Respir. J..

[16] Vertigan A.E., Kapela S.L., Ryan N.M., Birring S.S., McElduff P., Gibson P.G. (2016). Pregabalin and Speech Pathology Combination Therapy for Refractory Chronic Cough: A Randomized Controlled Trial. Chest.

[17] Ryan N.M., Birring S.S., Gibson P.G. (2012). Gabapentin for refractory chronic cough: A randomised, double-blind, placebo-controlled trial. Lancet.

[18] Morice A.H., Menon M.S., Mulrennan S.A., Everett C.F., Wright C., Jackson J., Thompson R. (2007). Opiate therapy in chronic cough. Am. J. Respir. Crit. Care Med..

[19] Jeyakumar A., Brickman T.M., Haben M. (2006). Effectiveness of amitriptyline versus cough suppressants in the treatment of chronic cough resulting from postviral vagal neuropathy. Laryngoscope.

[20] Gibson P., Wang G., McGarvey L., Vertigan A.E., Altman K.W., Birring S.S., Panel C.E.C. (2016). Treatment of Unexplained Chronic Cough: CHEST Guideline and Expert Panel Report. Chest.

[21] OneFlorida Clinical Research Consortium. https://www.ctsi.ufl.edu/ctsa-consortium-projects/oneflorida/.

[22] Hogan W.R., Shenkman E.A., Robinson T., Carasquillo O., Robinson P.S., Essner R.Z., Bian J., Lipori G., Harle C., Magoc T. (2022). The OneFlorida Data Trust: A centralized, translational research data infrastructure of statewide scope. J. Am. Med. Inform. Assoc..

[23] Fleurence R.L., Curtis L.H., Califf R.M., Platt R., Selby J.V., Brown J.S. (2014). Launching PCORnet, a national patient-centered clinical research network. J. Am. Med. Inform. Assoc..

[24] Bali V.W.J., Turzhitsky V., Schelfhout J., Paudel M., Hulbert E., Peterson-Brandt J., Hertzberg J., Kelly N.R., Patel R.H. (2021). Development of a Claims-Based Algorithm to Identify Patients with Chronic Cough. Am. J. Respir. Crit. Care Med..

[25] Weiner M., Dexter P.R., Heithoff K., Roberts A.R., Liu Z., Griffith A., Hui S., Schelfhout J., Dicpinigaitis P., Doshi I. (2021). Identifying and Characterizing a Chronic Cough Cohort Through Electronic Health Records. Chest.

[26] Bali V., Weaver J., Turzhitsky V., Schelfhout J., Paudel M.L., Hulbert E., Peterson-Brandt J., Currie A.G., Bakka D. (2022). Development of a natural language processing algorithm to detect chronic cough in electronic health records. BMC Pulm. Med..

[27] Zeiger R.S., Xie F., Schatz M., Hong B.D., Weaver J.P., Bali V., Schelfhout J., Chen W. (2020). Prevalence and Characteristics of Chronic Cough in Adults Identified by Administrative Data. Perm. J..

[28] Zhou L., Bhattacharjee S., Kwoh C.K., Tighe P.J., Malone D.C., Slack M., Wilson D.L., Brown J.D., Lo-Ciganic W.H. (2019). Trends, Patient and Prescriber Characteristics in Gabapentinoid Use in a Sample of United States Ambulatory Care Visits from 2003 to 2016. J. Clin. Med..

[29] Dowell D., Ragan K.R., Jones C.M., Baldwin G.T., Chou R. (2022). CDC Clinical Practice Guideline for Prescribing Opioids for Pain-United States, 2022. MMWR Recomm Rep..

[30] Meara E., Horwitz J.R., Powell W., McClelland L., Zhou W., O’Malley A.J., Morden N.E. (2016). State Legal Restrictions and Prescription-Opioid Use among Disabled Adults. N. Engl. J. Med..

[31] The National Patient-Centered Clinical Research Network Common Data Model (CDM) Specification, Version 6.0. https://pcornet.org/wp-content/uploads/2022/01/PCORnet-Common-Data-Model-v60-2020_10_221.pdf.

[32] Elixhauser A., Steiner C., Harris D.R., Coffey R.M. (1998). Comorbidity measures for use with administrative data. Med. Care.

[33] National Committee for Quality Assurance HEDIS Measures and Technical Resources. HEDIS MY 2021 Medication List. Directory. https://www.ncqa.org/hedis/measures/.

[34] Jones B.L., Nagin D.S. (2007). Advances in Group-Based Trajectory Modeling and an SAS Procedure for Estimating Them. Sociol. Methods Res..

[35] Nagin D.S., Jones B.L., Passos V.L., Tremblay R.E. (2018). Group-based multi-trajectory modeling. Stat. Methods Med. Res..

[36] Nagin D.S., Odgers C.L. (2010). Group-based trajectory modeling in clinical research. Annu. Rev. Clin. Psychol..

[37] Nagin D.S. (2005). Group-Based Modeling of Development.

[38] Twisk J., Hoekstra T. (2012). Classifying developmental trajectories over time should be done with great caution: A comparison between methods. J. Clin. Epidemiol..

[39] Yang S., Hincapie-Castillo J.M., Ke X., Schelfhout J., Ding H., Sher M.R., Zhou L., Chang C.Y., Wilson D.L., Lo-Ciganic W.H. (2022). Evaluation of Cough Medication Use Patterns in Ambulatory Care Settings in the United States: 2003–2018. J. Clin. Med..

[40] Kim I., Goulding M., Tian F., Karami S., Pham T., Cheng C., Biehl A., Munoz M. (2022). Benzonatate Exposure Trends and Adverse Events. Pediatrics.

[41] Smith R.V., Lofwall M.R., Havens J.R. (2015). Abuse and diversion of gabapentin among nonmedical prescription opioid users in Appalachian Kentucky. Am. J. Psychiatry.

[42] Smith R.V., Havens J.R., Walsh S.L. (2016). Gabapentin misuse, abuse and diversion: A systematic review. Addiction.

[43] Radley D.C., Finkelstein S.N., Stafford R.S. (2006). Off-label prescribing among office-based physicians. Arch. Intern. Med..

[44] Buttram M.E., Kurtz S.P., Dart R.C., Margolin Z.R. (2017). Law enforcement-derived data on gabapentin diversion and misuse, 2002–2015: Diversion rates and qualitative research findings. Pharmacoepidemiol. Drug Saf..

[45] Bonnet U., Scherbaum N. (2017). How addictive are gabapentin and pregabalin? A systematic review. Eur. Neuropsychopharmacol..

[46] National Center for Immunization and Respiratory Diseases (NCIRD), Division of Viral Diseases Symptoms of COVID-19. https://www.cdc.gov/coronavirus/2019-ncov/symptoms-testing/symptoms.html.

[47] Colak Y., Nordestgaard B.G., Laursen L.C., Afzal S., Lange P., Dahl M. (2017). Risk Factors for Chronic Cough Among 14,669 Individuals From the General Population. Chest.

[48] Won H.K., Kang S.Y., Kang Y., An J., Lee J.H., Lee S.M., Kwon J.W., Kim M.H., Jo E.J., Lee S.E. (2019). Cough-Related Laryngeal Sensations and Triggers in Adults With Chronic Cough: Symptom Profile and Impact. Allergy Asthma Immunol. Res..

[49] Hilton E., Marsden P., Thurston A., Kennedy S., Decalmer S., Smith J.A. (2015). Clinical features of the urge-to-cough in patients with chronic cough. Respir. Med..

[50] Arinze J.T., Verhamme K.M.C., Luik A.I., Stricker B., van Meurs J.B.J., Brusselle G.G. (2021). The interrelatedness of chronic cough and chronic pain. Eur. Respir. J..

[51] Arinze J.T., Hofman A., de Roos E.W., de Ridder M.A.J., Verhamme K.M.C., Stricker B., Brusselle G.G., Luik A.I. (2022). The interrelationship of chronic cough and depression: A prospective population-based study. ERJ Open Res..

[52] Sohn K.H., Song W.J., Kim S.H., Jang H.C., Kim K.W., Chang Y.S. (2019). Chronic cough, not asthma, is associated with depression in the elderly: A community-based population analysis in South Korea. Korean J. Intern. Med..

[53] Won H.K., Lee J.H., An J., Sohn K.H., Kang M.G., Kang S.Y., Morice A.H., Cho S.H., Song W.J. (2020). Impact of Chronic Cough on Health-Related Quality of Life in the Korean Adult General Population: The Korean National Health and Nutrition Examination Survey 2010–2016. Allergy Asthma Immunol. Res..

[54] Kardos P., Blaiss M., Dicpinigaitis P. (2021). Addressing unmet needs for diagnosis and management of chronic cough in the primary care setting. Postgrad. Med..

[55] Rouadi P.W., Idriss S.A., Bousquet J., Laidlaw T.M., Azar C.R., Al-Ahmad M.S., Yanez A., Al-Nesf M.A.Y., Nsouli T.M., Bahna S.L. (2022). WAO-ARIA consensus on chronic cough-Part III: Management strategies in primary and cough-specialty care. Updates in COVID-19. World Allergy Organ. J..

[56] Puente-Maestu L., Molina-Paris J., Trigueros J.A., Gomez-Saenz J.T., Cea-Calvo L., Fernandez S., Sanchez-Jareno M., Dominguez-Ortega J. (2021). A Survey of Physicians’ Perception of the Use and Effectiveness of Diagnostic and Therapeutic Procedures in Chronic Cough Patients. Lung.

[57] Leuppi J.D., Guggisberg P., Koch D., Favre-Bulle A., Fabiani M., Heinz S., Zeller A. (2022). Understanding physician’s knowledge and perception of chronic cough in Switzerland. Curr. Med. Res. Opin..

[58] Kum E., Brister D., Diab N., Wahab M., Abraham T., Sahakian S., Qureshy K., Hernandez P., Kim H., Cormier M. (2023). Canadian Health Care Professionals’ Familiarity with Chronic Cough Guidelines and Experiences with Diagnosis and Management: A Cross-Sectional Survey. Lung.

[59] Hull J.H., Langerman H., Ul-Haq Z., Kamalati T., Lucas A., Levy M.L. (2021). Burden and impact of chronic cough in UK primary care: A dataset analysis. BMJ Open.

