# Supplementary material for: Patterns of Cough Medication Prescribing among Patients with Chronic Cough in Florida: 2012–2021
Source: J Clin Med. 2023 Sep 29;12(19):6286. doi: 10.3390/jcm12196286 (PMC10573590; doi:10.3390/jcm12196286)
Supplement: Supplementary file 1 [file jcm-12-06286-s001.zip › jcm-2607559-supplementary.pdf]

Journal of
Clinical Medicine

MDPI

SUPPLEMENTARY MATERIALS

Patterns of Cough Medication Prescribing among Patients with Chronic Cough in Florida: 2012–2021

Seonkyeong Yang, Shu Huang, Juan M. Hincapie-Castillo, Xuehua Ke, Helen Ding, Jonathan Schelfhout, Mandel R. Sher, Bobby Jones, Debbie L. Wilson and Wei-Hsuan Lo-Ciganic

Table S1. ICD-9/10-CM codes to identify cough-related diagnosis

Table S2. Trends in annual cough medication (CM) prescribing prevalence in 2012-2021 OneFlorida data

Table S3. Pre-index characteristics of chronic cough (CC) patients and non-CC patients with any cough-related diagnosis

Table S4. Pre-index characteristics of chronic cough (CC) patients by cough medication (CM) utilization trajectory group

Table S5. Pre-index characteristics of non-chronic cough (CC) patients with any cough-related diagnosis by cough medication (CM) utilization trajectory group

Table S6. Post-index characteristics of chronic cough (CC) patients and non-CC patients with any cough-related diagnosis

Table S7. Prescribing specialty for all post-index cough medication (CM) prescription orders by CM utilization trajectory group

Table S8. Post-index characteristics of chronic cough (CC) patients by cough medication (CM) utilization trajectory group

Table S9. Post-index characteristics of non-chronic cough (CC) patients with any cough-related diagnosis by cough medication (CM) utilization trajectory group

Table S10. Odds ratios for pre-index factors associated with cough medication (CM) utilization trajectories in chronic cough (CC) patients

Figure S1. Chronic cough identification algorithm

Figure S2. Study design diagram for chronic cough (CC) patients in group-based trajectory modeling analysis

Figure S3. Study design diagram for non-chronic cough (CC) patients with any cough-related diagnosis in group-based trajectory modeling (GBTM) analysis

Journal of
Clinical Medicine

Figure S4. Flowchart for group-based trajectory modeling (GBTM) analysis: 2012−2021 OneFlorida data

Figure S5. Top 10 post-index respiratory comorbidities: 2012−2021 OneFlorida data

Figure S6. Top 10 post-index non-respiratory comorbidities: 2012−2021 OneFlorida data

Figure S7. Top 10 post-index concomitant medication use: 2012−2021 OneFlorida data

Figure S8. Post-index cough medication (CM) use: 2012−2021 OneFlorida data

Figure S9. Forest plot showing odds ratios for factors associated with cough medication (CM) utilization trajectories in chronic cough (CC) patients

Figure S10. Trajectories of opioid antitussive utilization: 2012−2021 OneFlorida data

Figure S11. Trajectories of benzonatate utilization: 2012−2021 OneFlorida data

Figure S12. Trajectories of dextromethorphan-containing medication utilization: 2012−2021 OneFlorida data

Figure S13. Trajectories of gabapentinoid utilization: 2012−2021 OneFlorida data

Figure S14. Trajectories of cough medication (CM) utilization including gabapetinoids: 2012−2021 OneFlorida data

Journal of
Clinical Medicine

Table S1. ICD-9/10-CM codes to identify cough-related diagnosis

Conditions	ICD-9-CM codes	ICD-10-CM codes
Acute URTI	460 461.x 462 464.0x 464.4 465.x	J00 J01.xx J02.x J04.0 J05.0 J06.x
Bronchitis	466.0 490 491.xx	J20.x J40 J41.x J42
Chronic URTD	472.x 473.x 476.0	J31.x J32.x J37.0
Cough	786.2	R05
Influenza	487.x 488.xx	J09.xx J10.xx J11.xx
Pneumonia	480.x 481 482.xx 483.x 484.x 485 486	J12.xx J13 J14 J15.xx J16.x J17 J18.x

Abbreviations: ICD-9/10-CM: International Classification of Diseases, Ninth/Tenth Revision, Clinical Modification; URTI: upper respiratory tract infection; URTD: upper respiratory tract diseases

Journal of
Clinical Medicine

Table S2. Trends in annual cough medication (CM) prescribing prevalence in 2012-2021 OneFlorida data

	2012	2013	2014	2015	2016	2017	2018	2019	2020	2021
CC patients, N	964	1,462	1,697	1,931	2,233	2,572	2,605	2,324	2,720	1,956
Opioid antitussives, %	8.4	8.5	13.2	14.7	13.8	13.8	10.3	10.0	3.8	6.7
Gabapentinoids, %	5.3	7.9	10.3	11.4	10.6	11.8	11.2	12.5	10.3	14.4
Benzonatate, %	9.6	12.2	14.6	21.3	20.5	18.4	20.8	19.4	19.5	26.1
Dextromethorphan, %	5.2	7.3	7.8	7.7	7.4	6.7	7.9	9.0	5.7	8.6
Opioid analgesics, %	21.3	25.8	30.1	31.4	28.1	24.8	25.1	20.6	14.2	18.6
Non-CC patients with any cough-related diagnosis, N	179,637	222,940	236,606	242,860	284,538	300,353	297,809	284,174	299,467	317,434
Opioid antitussives, %	1.6	1.8	2.5	2.8	3.0	3.0	2.4	1.6	0.7	1.2
Gabapentinoids, %	2.4	2.5	3.8	4.4	4.1	4.2	4.8	5.0	5.1	5.6
Benzonatate, %	2.3	2.5	3.9	4.5	5.5	5.9	6.8	6.0	5.5	7.1
Dextromethorphan, %	0.7	0.7	1.1	1.3	1.4	1.5	2.1	2.4	1.6	1.9
Opioid analgesics, %	13.9	14.0	18.1	18.1	16.2	14.8	13.7	10.8	9.3	10.8

Abbreviations: CC: chronic cough

Journal of
Clinical Medicine

Table S3. Pre-index characteristics of chronic cough (CC) patients and non-CC patients with any cough-related diagnosis

Pre-index characteristics [a]	All CC patients	All non-CC patients	p-value
N	15,566	655,260	
**Socio-demographics, %**			
Age, mean (SD)	53.9 (18.2)	46.3 (19.4)	<0.001
Female	70.2	68.6	<0.001
Race			<0.001
White	62.3	61.7	
Black	27.6	26.4	
Other	10.1	11.9	
Ethnicity			<0.001
Hispanic	21.4	23.8	
Non-Hispanic	76.6	73.7	
Unknown	2.0	2.5	
Payer			<0.001
Medicare	48.1	31.1	
Medicaid	38.3	40.5	
Commercial	9.0	19.1	
Other	4.6	9.3	
Residency			<0.001
Metropolitan counties	88.7	86.6	
Non-metropolitan counties	6.3	5.3	
Unknown	4.9	8.1	
**Healthcare service use, %**			
≥1 hospitalization	25.7	13.5	<0.001
No. of ED visits			<0.001
0	49.3	60.6	
1	17.8	21.9	
≥2	33.0	17.4	
No. of outpatient visits			<0.001
0	6.8	15.1	
1	8.3	20.3	
2-4	20.7	29.4	
≥5	64.2	35.2	
**Comorbidity index, mean (SD)**			
Elixhauser index [b]	1.7 (1.9)	0.7 (1.3)	<0.001
**Respiratory comorbidities, %**			
Acute URTI	19.6	0.0	<0.001
Airway malacia	0.1	0.0	<0.001
Allergic bronchopulmonary aspergillosis	0.1	0.0	<0.001
Allergic rhinitis	11.7	3.4	<0.001
Asthma	22.8	6.3	<0.001
Bronchiectasis	2.1	0.2	<0.001
Bronchiolitis obliterans	0.4	0.0	<0.001
Bronchitis	18.7	0.0	<0.001
Chronic URTD	7.1	0.0	<0.001
Chronic rhinitis	1.6	0.0	<0.001
Chronic sinusitis	5.7	0.0	<0.001

Journal of
Clinical Medicine

MDPI

Chronic tonsillitis	0.1	0.0	<0.001
COPD	28.0	4.8	<0.001
Costochondritis	1.0	0.3	<0.001
Cough	25.9	0.0	<0.001
COVID-19/coronavirus	0.9	0.4	<0.001
Cystic fibrosis	1.0	0.1	<0.001
Eosinophilic bronchitis/pneumonia	0.2	0.0	<0.001
Hemoptysis	2.0	0.4	<0.001
Hyperventilation	0.1	0.0	0.11
Influenza	1.7	0.0	<0.001
Nasal polyp disease	0.3	0.1	<0.001
Obstructive sleep apnea	9.6	3.1	<0.001
Pertussis	6.6	4.1	<0.001
Pneumonia	12.8	0.0	<0.001
Pulmonary fibrosis	3.1	0.4	<0.001
Respiratory foreign body	0.8	0.1	<0.001
Sarcoidosis	0.7	0.2	<0.001
SARS	*	0.0	0.51
Tonsillar enlargement	0.2	0.1	0.003
Upper airway cough syndrome	1.2	0.2	<0.001
Vocal cord dysfunction	0.3	0.1	<0.001
**Non-respiratory comorbidities, %**			
Alcohol use disorder	3.9	2.3	<0.001
Anxiety disorders	26.0	11.9	<0.001
Atrial fibrillation	7.4	3.4	<0.001
Coronary artery disease	28.9	16.8	<0.001
Eosinophilia	0.2	0.1	<0.001
Eosinophilic esophagitis/gastroenteritis	0.1	0.0	0.001
Gastroesophageal reflux disease	26.8	9.6	<0.001
Granulomatosis	0.1	0.0	<0.001
Heart failure	12.8	4.3	<0.001
HIV infection	3.0	1.2	<0.001
Hypertension	55.0	29.6	<0.001
Immune deficiency	1.0	0.2	<0.001
Immunosuppression treatments	0.0	0.0	N/A
Mast cell disease	0.4	0.1	<0.001
Mood disorders	27.9	13.0	<0.001
Musculoskeletal disorders	57.5	33.8	<0.001
Non-opioid substance use disorders	9.1	5.2	<0.001
Obesity	23.9	12.5	<0.001
Opioid use disorder	3.1	1.3	<0.001
Other immune disorders	5.4	2.4	<0.001
Peripheral vascular disease	5.2	2.2	<0.001
Relapsing polychondritis	0.2	0.1	0.001
Rib fracture	0.5	0.3	<0.001
Sleep disturbance	7.9	3.0	<0.001
Stress incontinence	2.3	0.7	<0.001
Subconjunctival hemorrhage	0.3	0.2	0.001
Transplantation of major organ	1.1	0.3	<0.001
Vomiting	5.0	1.9	<0.001
**Procedures, %**			

Journal of
Clinical Medicine

Allergy RAST testing	1.4	0.3	<0.001
Barium swallow or upper GI imaging	2.6	0.6	<0.001
Bronchoscopy	1.8	0.2	<0.001
Chest CT/MRI/ultrasound	13.3	2.9	<0.001
Chest X-ray	41.1	14.2	<0.001
Complete blood cell count	51.0	33.0	<0.001
Esophageal endoscopy	5.1	2.0	<0.001
Esophageal pH studies/manometry	0.3	0.1	<0.001
Laryngoscopy	1.4	0.4	<0.001
Methacholine challenge	*	*	0.02
Nasal/sinus endoscopy	1.2	0.3	<0.001
Sinus X-ray/CT	2.1	0.7	<0.001
Spirometry	6.5	1.0	<0.001
**Cough medications, %**			
Benzonatate	2.7	1.1	<0.001
Dextromethorphan	1.0	0.3	<0.001
Gabapentinoids	5.5	2.3	<0.001
Opioid antitussives	1.6	0.4	<0.001
**Cardiovascular medications, %**			
ACE inhibitors	6.8	3.9	<0.001
**Respiratory medications (oral, inhaled, or nasal), %**			
Biologics for asthma	0.1	0.0	<0.001
H1 antihistamines	9.6	4.8	<0.001
ICS monotherapy	1.8	0.3	<0.001
ICS/LABA combination	5.1	1.0	<0.001
ICS/LABA/LAMA combination	*	0.0	<0.001
LABA monotherapy	0.3	0.0	<0.001
LABA/LAMA combination	0.1	0.0	0.003
LAMA monotherapy	1.8	0.3	<0.001
Leukotriene modifiers	4.0	0.8	<0.001
Mast cell inhibitors inhaled	0.0	*	1.00
Mast cell inhibitors nasal	0.0	0.0	N/A
Mast cell inhibitors oral	0.0	0.0	N/A
Nasal antihistamines	0.5	0.3	<0.001
Nasal antihistamines/corticosteroids	0.1	0.0	0.002
Nasal corticosteroids	6.0	2.5	<0.001
Nasal SAMA	0.3	0.1	<0.001
PDE4 inhibitors for COPD	*	0.0	<0.001
SABA singly inhaled	10.9	2.7	<0.001
SABA/SAMA combination	5.0	1.0	<0.001
SAMA singly inhaled	2.3	0.3	<0.001
Theophylline	0.1	0.0	<0.001
**Gastrointestinal medications (oral), %**			
H2 antihistamines	4.6	2.5	<0.001
Proton pump inhibitors	11.4	4.6	<0.001
**Miscellaneous medications (oral), %**		0.3	
Antibiotics for respiratory conditions	19.4	1.0	<0.001
Corticosteroids	8.4	0.0	<0.001
**Pain medications, psychotherapeutics, others (oral), %**			
Antidepressants	10.8	5.1	<0.001
Antipsychotics	3.1	1.4	<0.001

Journal of
Clinical Medicine

Benzodiazepines	8.4	4.7	<0.001
Muscle relaxants	5.8	2.8	<0.001
Non-benzodiazepine hypnotics	1.8	1.0	<0.001
Opioid analgesics	13.7	8.2	<0.001
Other anxiolytics	0.9	0.3	<0.001
Other neuromodulators	4.5	2.2	<0.001
**Polypharmacy, %**			
≥3 different medications	28.1	18.7	<0.001

Abbreviations: CC: Chronic Cough; CM: Cough Medication; SD: Standard Deviation; ED: Emergency Department; URTI: Upper Respiratory Tract Infection; URTD: Upper Respiratory Tract Disease; COPD: Chronic Obstructive Pulmonary Disease; COVID-19: Coronavirus Disease caused by the SARS-CoV-2 virus; HIV: Human Immunodeficiency Virus; RAST: Radioallergosorbent Test; GI: Gastrointestinal; CT: Computerized Tomography; MRI: Magnetic Resonance Imaging; ACE: Angiotensin Converting Enzyme; H1: Histamine-1 receptor; ICS: Inhaled Corticosteroid; LABA: Long-acting Beta-agonist; LAMA: Long-acting Muscarinic-antagonist; PDE-4: Phosphodiesterase-4; SAMA: Short-acting Muscarinic-antagonist; SABA: Short-acting Beta agonist; H2: Histamine-2 receptor; HIPAA: Health Insurance Portability and Accountability Act. * A percentage from a count of patients less than 11 or used to derive a count of patients less than 11 in other cells is not reportable according to HIPAA Privacy Rule. [a] Pre-index period is 6 months before the index date. [b] Modified Elixhauser Comorbidity Index was calculated by excluding metastatic cancers, solid tumors with or without metastasis, and diseases/conditions examined individually.

Journal of
Clinical Medicine

MDPI

Table S4. Pre-index characteristics of chronic cough (CC) patients by cough medication (CM) utilization trajectory group [a]

Pre-index characteristics [b]	All CC patients	Group 1 (No CM use)	Group 2 (Declining CM use)	Group 3 (Chronic CM use)	p-value
N	15,566	11,222	4,105	239	
**Socio-demographics, %**					
Age, mean (SD)	53.9 (18.2)	53.6 (18.8)	54.3 (16.6)	58.1 (13.5)	<0.001
Female	70.2	69.4	72.6	70.7	0.002
Race					<0.001
White	62.3	62.8	61.5	55.7	
Black	27.6	26.6	29.7	36.8	
Other	10.1	10.7	8.8	7.5	
Ethnicity					<0.001
Hispanic	21.4	23.8	15.3	*	
Non-Hispanic	76.6	74.2	82.7	85.8	
Unknown	2.0	2.1	2.0	*	
Payer					<0.001
Medicare	48.1	47.7	48.6	56.9	
Medicaid	38.3	43.9	24.4	16.3	
Commercial	9.0	4.3	21.0	22.2	
Other	4.6	4.1	6.1	4.6	
Residency					<0.001
Metropolitan counties	88.7	89.1	87.6	90.8	
Non-metropolitan counties	6.3	5.8	7.7	7.1	
Unknown	4.9	5.1	4.7	2.1	
**Healthcare service use, %**					
≥1 hospitalization	25.7	26.4	23.6	31.0	<0.001
No. of ED visits					<0.001
0	49.3	46.9	55.5	54.4	
1	17.8	18.2	16.8	14.2	
≥2	33.0	34.9	27.7	31.4	
No. of outpatient visits					<0.001
0	6.8	6.4	8.0	8.4	
1	8.3	7.9	9.4	7.1	
2-4	20.7	20.4	21.7	13.0	
≥5	64.2	65.3	61.0	71.6	
**Comorbidity index, mean (SD)**					
Elixhauser index [c]	1.7 (1.9)	1.7 (1.9)	1.4 (1.8)	1.8 (2.0)	<0.001
**Respiratory comorbidities, %**					
Acute URTI	19.6	19.6	19.6	19.7	1.00
Airway malacia	0.1	*	*	*	0.07
Allergic bronchopulmonary aspergillosis	0.1	*	*	0.0	1.00
Allergic rhinitis	11.7	11.3	12.7	16.3	0.005
Asthma	22.8	22.5	23.1	32.6	0.001
Bronchiectasis	2.1	2.1	2.1	*	0.38
Bronchiolitis obliterans	0.4	0.4	0.3	0.0	0.59
Bronchitis	18.7	18.8	17.9	25.9	0.007
Chronic URTD	7.1	7.4	5.9	13.8	<0.001
Chronic rhinitis	1.6	1.7	1.4	*	0.34
Chronic sinusitis	5.7	5.9	4.7	12.1	<0.001

Journal of
Clinical Medicine

MDPI

Chronic tonsillitis	0.1	0.1	*	0.0	1.00
COPD	28.0	29.5	23.4	36.0	<0.001
Costochondritis	1.0	1.0	1.0	*	0.06
Cough	25.9	27.4	21.5	29.3	<0.001
COVID-19/coronavirus	0.9	1.0	0.6	*	0.03
Cystic fibrosis	1.0	1.2	0.5	0.0	<0.001
Eosinophilic bronchitis/pneumonia	0.2	0.2	*	0.0	0.32
Hemoptysis	2.0	2.0	1.8	*	0.68
Hyperventilation	0.1	*	*	0.0	0.78
Influenza	1.7	1.8	1.5	*	0.10
Nasal polyp disease	0.3	0.3	*	*	0.45
Obstructive sleep apnea	9.6	8.8	11.5	14.2	<0.001
Pertussis	6.6	6.7	6.2	7.5	0.48
Pneumonia	12.8	13.6	10.5	11.7	<0.001
Pulmonary fibrosis	3.1	2.7	4.0	6.3	<0.001
Respiratory foreign body	0.8	0.9	0.4	0.0	0.002
Sarcoidosis	0.7	0.6	0.8	*	0.34
SARS	*	*	0.0	0.0	0.59
Tonsillar enlargement	0.2	0.1	*	0.0	0.57
Upper airway cough syndrome	1.2	1.1	1.5	*	0.21
Vocal cord dysfunction	0.3	0.2	0.4	0.0	0.22
**Non-respiratory comorbidities, %**					
Alcohol use disorder	3.9	3.9	3.8	4.6	0.82
Anxiety disorders	26.0	26.1	25.1	33.9	0.008
Atrial fibrillation	7.4	7.4	7.3	8.8	0.68
Coronary artery disease	28.9	28.7	28.8	37.2	0.02
Eosinophilia	0.2	0.2	*	*	0.10
Eosinophilic esophagitis/gastroenteritis	0.1	*	*	*	0.22
Gastroesophageal reflux disease	26.8	26.7	26.5	34.7	0.02
Granulomatosis	0.1	0.1	*	0.0	0.89
Heart failure	12.8	13.1	11.7	16.7	0.02
HIV infection	3.0	3.3	2.2	*	0.001
Hypertension	55.0	55.2	54.2	60.7	0.11
Immune deficiency	1.0	1.0	0.9	*	0.01
Immunosuppression treatments	0.0	0.0	0.0	0.0	N/A
Mast cell disease	0.4	0.4	0.4	*	1.00
Mood disorders	27.9	29.5	23.8	23.4	<0.001
Musculoskeletal disorders	57.5	58.0	55.7	63.6	0.006
Non-opioid substance use disorders	9.1	9.5	8.1	10.5	0.03
Obesity	23.9	22.6	27.0	35.2	<0.001
Opioid use disorder	3.1	3.2	2.5	*	0.05
Other immune disorders	5.4	5.4	5.4	6.3	0.82
Peripheral vascular disease	5.2	5.6	4.3	*	0.008
Relapsing polychondritis	0.2	0.1	*	0.0	0.46
Rib fracture	0.5	0.5	0.5	*	0.90
Sleep disturbance	7.9	7.7	8.1	17.6	<0.001
Stress incontinence	2.3	2.1	2.6	*	0.19
Subconjunctival hemorrhage	0.3	0.3	0.4	*	0.56
Transplantation of major organ	1.1	0.9	1.5	*	0.001
Vomiting	5.0	5.3	4.2	*	0.008
**Procedures, %**					

Allergy RAST testing	1.4	1.6	0.9	*	0.003
Barium swallow or upper GI imaging	2.6	2.8	2.1	*	0.04
Bronchoscopy	1.8	1.8	1.9	*	0.37
Chest CT/MRI/ultrasound	13.3	13.7	11.9	14.2	0.02
Chest X-ray	41.1	45.0	31.0	32.2	<0.001
Complete blood cell count	51.0	55.6	39.0	37.2	<0.001
Esophageal endoscopy	5.1	5.6	3.9	*	<0.001
Esophageal pH studies/manometry	0.3	0.3	0.4	0.0	0.17
Laryngoscopy	1.4	1.4	1.3	*	0.60
Methacholine challenge	*	*	0.0	0.0	1.00
Nasal/sinus endoscopy	1.2	1.3	0.9	*	0.07
Sinus X-ray/CT	2.1	2.4	1.2	*	<0.001
Spirometry	6.5	7.1	5.2	*	<0.001
**Cough medications, %**					
Benzonatate	2.7	0.8	7.3	15.9	<0.001
Dextromethorphan	1.0	0.3	2.5	8.0	<0.001
Gabapentinoids	5.5	2.9	11.7	19.3	<0.001
Opioid antitussives	1.6	0.4	4.1	15.9	<0.001
**Cardiovascular medications, %**					
ACE inhibitors	6.8	4.0	13.7	19.7	<0.001
**Respiratory medications (oral, inhaled, or nasal), %**					
Biologics for asthma	0.1	*	*	*	<0.001
H1 antihistamines	9.6	5.1	20.7	32.2	<0.001
ICS monotherapy	1.8	1.2	3.3	6.3	<0.001
ICS/LABA combination	5.1	2.9	10.4	19.7	<0.001
ICS/LABA/LAMA combination	*	*	*	0.0	0.74
LABA monotherapy	0.3	0.1	0.6	*	<0.001
LABA/LAMA combination	0.1	*	*	0.0	0.10
LAMA monotherapy	1.8	0.9	4.0	10.0	<0.001
Leukotriene modifiers	4.0	2.2	8.1	15.9	<0.001
Mast cell inhibitors inhaled	0.0	0.0	0.0	0.0	N/A
Mast cell inhibitors nasal	0.0	0.0	0.0	0.0	N/A
Mast cell inhibitors oral	0.0	0.0	0.0	0.0	N/A
Nasal antihistamines	0.5	0.4	1.0	*	<0.001
Nasal antihistamines/corticosteroids	0.1	*	*	0.0	0.04
Nasal corticosteroids	6.0	3.1	13.0	20.9	<0.001
Nasal SAMA	0.3	0.1	0.7	0.0	<0.001
PDE4 inhibitors for COPD	*	*	*	*	0.05
SABA singly inhaled	10.9	6.3	22.2	33.9	<0.001
SABA/SAMA combination	5.0	2.7	10.5	21.8	<0.001
SAMA singly inhaled	2.3	1.3	4.6	12.6	<0.001
Theophylline	0.1	*	0.3	0.0	<0.001
**Gastrointestinal medications (oral), %**					
H2 antihistamines	4.6	2.8	8.9	13.0	<0.001
Proton pump inhibitors	11.4	6.8	23.1	30.1	<0.001
**Miscellaneous medications (oral), %**					
Antibiotics for respiratory conditions	19.4	10.7	41.1	59.4	<0.001
Corticosteroids	8.4	4.2	18.6	31.0	<0.001
**Pain medications, psychotherapeutics, others (oral), %**					
Antidepressants	10.8	5.9	23.1	32.2	<0.001
Antipsychotics	3.1	2.0	5.7	8.4	<0.001

Benzodiazepines	8.4	4.9	16.6	33.5	<0.001
Muscle relaxants	5.8	2.8	12.7	26.4	<0.001
Non-benzodiazepine hypnotics	1.8	0.9	3.7	10.0	<0.001
Opioid analgesics	13.7	7.6	28.4	50.6	<0.001
Other anxiolytics	0.9	0.5	1.8	*	<0.001
Other neuromodulators	4.5	2.7	8.8	10.9	<0.001
**Polypharmacy, %**					
≥3 different medications	28.1	17.2	55.3	74.1	<0.001

Abbreviations: CC: Chronic Cough; CM: Cough Medication; SD: Standard Deviation; ED: Emergency Department; URTI: Upper Respiratory Tract Infection; URTD: Upper Respiratory Tract Disease; COPD: Chronic Obstructive Pulmonary Disease; COVID-19: Coronavirus Disease caused by the SARS-CoV-2 virus; HIV: Human Immunodeficiency Virus; RAST: Radioallergosorbent Test; GI: Gastrointestinal; CT: Computerized Tomography; MRI: Magnetic Resonance Imaging; ACE: Angiotensin Converting Enzyme; H1: Histamine-1 receptor; ICS: Inhaled Corticosteroid; LABA: Long-acting Beta-agonist; LAMA: Long-acting Muscarinic-antagonist; PDE-4: Phosphodiesterase-4; SAMA: Short-acting Muscarinic-antagonist; SABA: Short-acting Beta agonist; H2: Histamine-2 receptor; HIPAA: Health Insurance Portability and Accountability Act. * A percentage from a count of patients less than 11 or used to derive a count of patients less than 11 in other cells is not reportable according to HIPAA Privacy Rule. [a] Distinct trajectory of any CM use (i.e., opioid antitussives, benzonatate, and dextromethorphan-containing medications), excluding gabapentinoids. [b] Pre-index period is 6 months before the index date. [c] Modified Elixhauser Comorbidity Index was calculated by excluding metastatic cancers, solid tumors with or with-out metastasis, and diseases/conditions examined individually.

Journal of Clinical Medicine

MDPI

Table S5. Pre-index characteristics of non-chronic cough (CC) patients with any cough-related diagnosis by cough medication (CM) utilization trajectory group [a]

Pre-index characteristics [b]	All non-CC patients	Group 1 (No CM use)	Group 2 (Declining CM use)	Group 3 (Chronic minimal CM use)	p-value
N	655,260	611,496	31,805	11,959	
**Socio-demographics, %**					
Age, mean (SD)	46.3 (19.4)	46.1 (19.4)	48.3 (18.2)	48.6 (18.1)	<0.001
Female	68.6	68.5	68.6	71.4	<0.001
Race					<0.001
White	61.7	61.6	62.6	65.6	
Black	26.4	26.5	25.7	23.9	
Other	11.9	11.9	11.7	10.5	
Ethnicity					<0.001
Hispanic	23.8	24.3	15.9	19.8	
Non-Hispanic	73.7	73.3	80.7	76.8	
Unknown	2.5	2.5	3.4	3.4	
Payer					<0.001
Medicare	31.1	31.1	30.0	32.6	
Medicaid	40.5	42.0	19.8	20.8	
Commercial	19.1	17.5	41.9	40.0	
Other	9.3	9.4	8.3	6.6	
Residency					<0.001
Metropolitan counties	86.6	86.5	88.3	88.6	
Non-metropolitan counties	5.3	5.2	7.0	7.3	
Unknown	8.1	8.4	4.7	4.1	
**Healthcare service use, %**					
≥1 hospitalization	13.5	13.8	9.1	10.8	<0.001
No. of ED visits					<0.001
0	60.6	60.2	66.3	68.4	
1	21.9	22.1	20.8	17.6	
≥2	17.4	17.7	12.9	14.0	
No. of outpatient visits					<0.001
0	15.1	14.8	20.9	15.1	
1	20.3	20.3	20.2	19.5	
2-4	29.4	29.4	29.7	29.6	
≥5	35.2	35.5	29.1	35.8	
**Comorbidity index, mean (SD)**					
Elixhauser index [c]	0.7 (1.3)	0.8 (1.3)	0.6 (1.1)	0.7 (1.2)	<0.001
**Respiratory comorbidities, % [d]**					
Airway malacia	0.0	0.0	*	*	N/A
Allergic bronchopulmonary aspergillosis	0.0	0.0	*	*	0.02
Allergic rhinitis	3.4	3.3	3.7	4.9	<0.001
Asthma	6.3	6.3	5.8	8.3	<0.001
Bronchiectasis	0.2	0.2	0.1	0.3	<0.001
Bronchiolitis obliterans	0.0	0.0	0.0	*	0.48
COPD	4.8	4.9	2.8	5.5	<0.001
Costochondritis	0.3	0.3	0.3	0.3	0.69
COVID-19/coronavirus	0.4	0.4	0.5	0.2	0.001
Cystic fibrosis	0.1	0.1	0.1	0.1	0.12

Journal of
Clinical Medicine

Eosinophilic bronchitis/pneumonia	0.0	0.0	*	*	0.05
Hemoptysis	0.4	0.4	0.3	0.3	0.001
Hyperventilation	0.0	0.0	*	*	0.14
Nasal polyp disease	0.1	0.1	*	*	<0.001
Obstructive sleep apnea	3.1	3.1	3.6	4.1	<0.001
Pertussis	4.1	4.1	3.0	4.1	<0.001
Pneumonia	0.0	0.0	0.0	0.0	N/A
Pulmonary fibrosis	0.4	0.4	0.4	0.9	<0.001
Respiratory foreign body	0.1	0.1	0.1	*	0.006
Sarcoidosis	0.2	0.2	0.2	0.2	0.09
SARS	0.0	0.0	*	0.0	0.08
Tonsillar enlargement	0.1	0.1	0.1	0.1	0.08
Upper airway cough syndrome	0.2	0.2	0.2	0.2	0.37
Vocal cord dysfunction	0.1	0.1	0.0	*	0.18
**Non-respiratory comorbidities, %**					
Alcohol use disorder	2.3	2.3	1.9	1.8	<0.001
Anxiety disorders	11.9	12.0	11.0	12.7	<0.001
Atrial fibrillation	3.4	3.4	3.2	3.4	0.06
Coronary artery disease	16.8	16.9	15.0	17.6	<0.001
Eosinophilia	0.1	0.1	*	*	0.23
Eosinophilic esophagitis/gastroenteritis	0.0	0.0	0.0	*	0.24
Gastroesophageal reflux disease	9.6	9.6	9.9	11.6	<0.001
Granulomatosis	0.0	0.0	*	*	0.43
Heart failure	4.3	4.4	3.1	4.6	<0.001
HIV infection	1.2	1.2	0.5	0.7	<0.001
Hypertension	29.6	29.4	31.5	32.6	<0.001
Immune deficiency	0.2	0.2	0.1	0.2	0.008
Immunosuppression treatments	0.0	0.0	0.0	0.0	N/A
Mast cell disease	0.1	0.1	0.1	0.1	<0.001
Mood disorders	13.0	13.2	10.0	11.2	<0.001
Musculoskeletal disorders	33.8	33.9	32.2	35.1	<0.001
Non-opioid substance use disorders	5.2	5.3	3.7	3.8	<0.001
Obesity	12.5	12.4	14.4	15.1	<0.001
Opioid use disorder	1.3	1.4	0.8	0.8	<0.001
Other immune disorders	2.4	2.4	1.8	2.5	<0.001
Peripheral vascular disease	2.2	2.3	1.4	1.9	<0.001
Relapsing polychondritis	0.1	0.1	0.1	0.1	0.23
Rib fracture	0.3	0.3	0.2	0.2	0.001
Sleep disturbance	3.0	3.0	3.7	4.3	<0.001
Stress incontinence	0.7	0.7	0.7	0.9	0.01
Subconjunctival hemorrhage	0.2	0.2	0.2	0.2	0.33
Transplantation of major organ	0.3	0.3	0.1	0.3	<0.001
Vomiting	1.9	1.9	1.6	1.7	<0.001
**Procedures, %**					
Allergy RAST testing	0.3	0.3	0.1	0.1	<0.001
Barium swallow or upper GI imaging	0.6	0.6	0.4	0.6	<0.001
Bronchoscopy	0.2	0.2	0.1	0.1	0.008
Chest CT/MRI/ultrasound	2.9	3.0	2.1	3.0	<0.001
Chest X-ray	14.2	14.5	9.1	11.7	<0.001
Complete blood cell count	33.0	33.9	18.6	22.6	<0.001
Esophageal endoscopy	2.0	2.0	1.4	1.6	<0.001

Journal of
Clinical Medicine

MDPI

Esophageal pH studies/manometry	0.1	0.1	*	*	0.01
Laryngoscopy	0.4	0.4	0.3	0.5	<0.001
Methacholine challenge	*	*	0.0	0.0	0.75
Nasal/sinus endoscopy	0.3	0.3	0.1	0.2	<0.001
Sinus X-ray/CT	0.7	0.7	0.3	0.5	<0.001
Spirometry	1.0	1.0	0.5	1.1	<0.001
**Cough medications, %**					
Benzonatate	1.1	1.0	1.9	6.1	<0.001
Dextromethorphan	0.3	0.2	0.6	1.7	<0.001
Gabapentinoids	2.3	2.1	4.4	5.1	<0.001
Opioid antitussives	0.4	0.3	0.8	1.8	<0.001
**Cardiovascular medications, %**					
ACE inhibitors	3.9	3.5	9.3	9.5	<0.001
**Respiratory medications (oral, inhaled, or nasal), %**					
Biologics for asthma	0.0	0.0	*	*	0.003
H1 antihistamines	4.8	4.5	8.4	12.0	<0.001
ICS monotherapy	0.3	0.3	0.5	1.0	<0.001
ICS/LABA combination	1.0	1.0	1.6	3.3	<0.001
ICS/LABA/LAMA combination	0.0	0.0	0.0	*	0.04
LABA monotherapy	0.0	0.0	0.0	0.1	<0.001
LABA/LAMA combination	0.0	0.0	*	0.1	<0.001
LAMA monotherapy	0.3	0.2	0.3	0.8	<0.001
Leukotriene modifiers	0.8	0.8	1.5	2.8	<0.001
Mast cell inhibitors inhaled	*	*	0.0	0.0	0.97
Mast cell inhibitors nasal	0.0	0.0	*	*	0.83
Mast cell inhibitors oral	0.0	0.0	0.0	0.0	0.56
Nasal antihistamines	0.3	0.3	0.3	0.9	<0.001
Nasal antihistamines/corticosteroids	0.0	0.0	0.1	*	0.07
Nasal corticosteroids	2.5	2.3	3.6	8.6	<0.001
Nasal SAMA	0.1	0.1	0.1	0.2	<0.001
PDE4 inhibitors for COPD	0.0	0.0	*	*	0.001
SABA singly inhaled	2.7	2.5	4.7	8.5	<0.001
SABA/SAMA combination	1.0	1.0	1.5	2.5	<0.001
SAMA singly inhaled	0.3	0.3	0.4	0.8	<0.001
Theophylline	0.0	0.0	*	*	<0.001
Biologics for asthma	0.0	0.0	*	*	0.003
H1 antihistamines	4.8	4.5	8.4	12.0	<0.001
ICS monotherapy	0.3	0.3	0.5	1.0	<0.001
ICS/LABA combination	1.0	1.0	1.6	3.3	<0.001
ICS/LABA/LAMA combination	0.0	0.0	0.0	*	0.04
**Gastrointestinal medications (oral), %**					
H2 antihistamines	2.5	2.3	4.5	5.1	<0.001
Proton pump inhibitors	4.6	4.3	8.9	11.2	<0.001
**Miscellaneous medications (oral), %**					
Antibiotics for respiratory conditions	10.6	9.9	19.1	26.8	<0.001
Corticosteroids	2.4	2.2	4.8	7.2	<0.001
**Pain medications, psychotherapeutics, others (oral), %**					
Antidepressants	5.1	4.7	11.4	13.5	<0.001
Antipsychotics	1.4	1.4	2.1	2.5	<0.001
Benzodiazepines	4.7	4.5	7.9	9.9	<0.001
Muscle relaxants	2.8	2.5	6.8	7.6	<0.001

Non-benzodiazepine hypnotics	1.0	0.9	2.2	2.9	<0.001
Opioid analgesics	8.2	7.5	16.8	17.7	<0.001
Other anxiolytics	0.3	0.3	0.7	0.9	<0.001
Other neuromodulators	2.2	2.1	3.9	5.3	<0.001
**Polypharmacy, %**					
≥3 different medications	18.7	17.6	31.1	42.8	<0.001

Abbreviations: CC: Chronic Cough; CM: Cough Medication; SD: Standard Deviation; ED: Emergency Department; URTI: Upper Respiratory Tract Infection; URTD: Upper Respiratory Tract Disease; COPD: Chronic Obstructive Pulmonary Disease; COVID-19: Coronavirus Disease caused by the SARS-CoV-2 virus; HIV: Human Immunodeficiency Virus; RAST: Radioallergosorbent Test; GI: Gastrointestinal; CT: Computerized Tomography; MRI: Magnetic Resonance Imaging; ACE: Angiotensin Converting Enzyme; H1: Histamine-1 receptor; ICS: Inhaled Corticosteroid; LABA: Long-acting Beta-agonist; LAMA: Long-acting Muscarinic-antagonist; PDE-4: Phosphodiesterase-4; SAMA: Short-acting Muscarinic-antagonist; SABA: Short-acting Beta agonist; H2: Histamine-2 receptor; HIPAA: Health Insurance Portability and Accountability Act. * A percentage from a count of patients less than 11 or used to derive a count of patients less than 11 in other cells is not reportable according to HIPAA Privacy Rule. [a] Distinct trajectory of any CM use (i.e., opioid antitussives, benzonatate, and dextromethorphan-containing medications), excluding gabapentinoids. [b] Pre-index period is 6 months before the index date. [c] Modified Elixhauser Comorbidity Index was calculated by excluding metastatic cancers, solid tumors with or with-out metastasis, and diseases/conditions examined individually. [d] Due to the definition of index date for non-CC patients with any cough-related diagnosis, prevalence of acute URTI, bronchitis, chronic URTD, cough, influenza, and pneumonia is 0% during the pre-index period.

Journal of
Clinical Medicine

MDPI

Table S6. Post-index characteristics of chronic cough (CC) patients and non-CC patients with any cough-related diagnosis

Post-index characteristics [a]	All CC patients	All non-CC patients	p-value
N	15,566	655,260	
**Healthcare service use, %**			
≥1 hospitalization	41.1	28.1	<0.001
No. of ED visits			<0.001
0	31.9	41.8	
1	13.7	19.9	
≥2	54.4	38.2	
No. of outpatient visits			<0.001
0	2.2	9.2	
1	1.3	6.7	
2-4	4.3	22.0	
≥5	92.2	62.1	
**Comorbidity index, mean (SD)**			
Elixhauser index [b]	2.7 (2.5)	1.3 (1.8)	<0.001
**Number of visits with cough-related diagnosis, mean (SD)**			
No. of visits with acute URTI	1.9 (3.8)	1.2 (2.0)	<0.001
No. of visits with bronchitis	2.5 (6.6)	0.6 (1.8)	<0.001
No. of visits with chronic URTD	1.0 (5.8)	0.4 (2.1)	<0.001
No. of visits with cough	9.1 (9.0)	0.9 (1.8)	<0.001
No. of visits with influenza	0.3 (1.6)	0.1 (0.9)	<0.001
No. of visits with pneumonia	3.5 (13.7)	0.7 (3.9)	<0.001
No. of visits with any cough-related Dx	18.3 (21.1)	3.8 (5.7)	<0.001
**Respiratory comorbidities, %**			
Acute URTI	44.7	49.4	<0.001
Airway malacia	0.2	1.4	<0.001
Allergic bronchopulmonary aspergillosis	0.2	0.0	<0.001
Allergic rhinitis	28.2	9.5	<0.001
Asthma	40.8	13.3	<0.001
Bronchiectasis	5.2	0.5	<0.001
Bronchiolitis obliterans	1.2	0.2	<0.001
Bronchitis	45.7	22.4	<0.001
Chronic URTD	18.1	12.8	<0.001
Chronic rhinitis	5.4	2.5	<0.001
Chronic sinusitis	13.9	9.8	<0.001
Chronic tonsillitis	0.3	0.2	0.02
COPD	50.8	18.3	<0.001
Costochondritis	2.8	0.9	<0.001
Cough	98.2	39.2	<0.001
COVID-19/coronavirus	7.7	3.3	<0.001
Cystic fibrosis	1.3	0.2	<0.001
Eosinophilic bronchitis/pneumonia	0.7	0.1	<0.001
Hemoptysis	5.9	1.6	<0.001
Hyperventilation	0.3	0.1	<0.001
Influenza	6.8	6.1	<0.001
Nasal polyp disease	0.8	0.4	<0.001
Obstructive sleep apnea	18.4	6.1	<0.001
Pertussis	8.8	6.5	<0.001

Journal of
Clinical Medicine

MDPI

Pneumonia	30.6	14.6	<0.001
Pulmonary fibrosis	7.3	1.3	<0.001
Respiratory foreign body	2.4	0.5	<0.001
Sarcoidosis	1.2	0.3	<0.001
SARS	0.1	0.1	<0.001
Tonsillar enlargement	0.5	0.4	0.005
Upper airway cough syndrome	6.3	1.4	<0.001
Vocal cord dysfunction	1.2	0.2	<0.001
**Non-respiratory comorbidities, %**			
Alcohol use disorder	6.0	3.7	<0.001
Anxiety disorders	39.5	20.0	<0.001
Atrial fibrillation	11.6	6.0	<0.001
Coronary artery disease	39.6	24.3	<0.001
Eosinophilia	0.9	0.2	<0.001
Eosinophilic esophagitis/gastroenteritis	0.2	0.1	<0.001
Gastroesophageal reflux disease	48.8	18.3	<0.001
Granulomatosis	0.2	0.1	<0.001
Heart failure	21.5	8.7	<0.001
HIV infection	3.6	1.6	<0.001
Hypertension	68.0	40.7	<0.001
Immune deficiency	2.3	0.7	<0.001
Immunosuppression treatments	*	0.0	0.01
Mast cell disease	1.0	0.3	<0.001
Mood disorders	39.8	20.2	<0.001
Musculoskeletal disorders	75.7	48.4	<0.001
Non-opioid substance use disorders	14.9	8.6	<0.001
Obesity	36.5	20.9	<0.001
Opioid use disorder	5.5	2.4	<0.001
Other immune disorders	8.7	4.1	<0.001
Peripheral vascular disease	9.5	3.9	<0.001
Relapsing polychondritis	0.3	0.2	<0.001
Rib fracture	1.3	0.6	<0.001
Sleep disturbance	15.2	5.7	<0.001
Stress incontinence	4.1	1.4	<0.001
Subconjunctival hemorrhage	0.9	0.4	<0.001
Transplantation of major organ	1.7	0.6	<0.001
Vomiting	10.9	4.2	<0.001
**Procedures, %**			
Allergy RAST testing	5.3	0.7	<0.001
Barium swallow or upper GI imaging	8.3	1.9	<0.001
Bronchoscopy	5.9	1.1	<0.001
Chest CT/MRI/ultrasound	35.2	9.5	<0.001
Chest X-ray	74.6	39.7	<0.001
Complete blood cell count	68.5	47.3	<0.001
Esophageal endoscopy	11.9	4.4	<0.001
Esophageal pH studies/manometry	1.4	0.2	<0.001
Laryngoscopy	6.9	1.3	<0.001
Methacholine challenge	0.2	0.0	<0.001
Nasal/sinus endoscopy	3.8	1.6	<0.001
Sinus X-ray/CT	7.5	2.9	<0.001
Spirometry	24.6	3.4	<0.001

**Cough medications**			
Benzonatate, %	19.7	4.4	<0.001
Dextromethorphan, %	7.7	1.2	<0.001
Gabapentinoids, %	10.3	3.9	<0.001
Opioid antitussives, %	11.2	2.1	<0.001
No. of any CM prescriptions including gabapentinoids, mean (SD)	1.1 (2.3)	0.2 (0.7)	<0.001
No. of any CM prescriptions excluding gabapentinoids, mean (SD)	0.8 (1.6)	0.1 (0.4)	<0.001
**Cardiovascular medications, %**			
ACE inhibitors	8.9	5.4	<0.001
**Respiratory medications (oral, inhaled, or nasal), %**			
Biologics for asthma	0.3	0.0	<0.001
H1 antihistamines	20.6	9.0	<0.001
ICS monotherapy	5.7	0.8	<0.001
ICS/LABA combination	13.5	2.3	<0.001
ICS/LABA/LAMA combination	0.2	0.0	<0.001
LABA monotherapy	0.8	0.1	<0.001
LABA/LAMA combination	0.3	0.1	<0.001
LAMA monotherapy	4.6	0.7	<0.001
Leukotriene modifiers	10.0	1.7	<0.001
Mast cell inhibitors inhaled	0.0	*	1.00
Mast cell inhibitors nasal	*	0.0	0.16
Mast cell inhibitors oral	*	0.0	0.09
Nasal antihistamines	1.9	0.5	<0.001
Nasal antihistamines/corticosteroids	0.2	0.1	<0.001
Nasal corticosteroids	15.3	5.2	<0.001
Nasal SAMA	1.0	0.2	<0.001
PDE4 inhibitors for COPD	0.3	0.0	<0.001
SABA singly inhaled	26.1	7.5	<0.001
SABA/SAMA combination	12.3	3.4	<0.001
SAMA singly inhaled	5.4	0.9	<0.001
Theophylline	0.4	0.0	<0.001
**Gastrointestinal medications (oral), %**			
H2 antihistamines	10.6	4.6	<0.001
Proton pump inhibitors	22.1	7.7	<0.001
**Miscellaneous medications (oral), %**			
Antibiotics for respiratory conditions	35.9	24.6	<0.001
Corticosteroids	20.8	7.2	<0.001
**Pain medications, psychotherapeutics, others (oral), %**			
Antidepressants	17.3	7.8	<0.001
Antipsychotics	6.1	2.6	<0.001
Benzodiazepines	14.4	7.7	<0.001
Muscle relaxants	10.7	4.9	<0.001
Non-benzodiazepine hypnotics	3.1	1.5	<0.001
Opioid analgesics	22.2	13.7	<0.001
Other anxiolytics	1.8	0.6	<0.001
Other neuromodulators	7.4	3.5	<0.001
**Polypharmacy, %**			
≥3 different medications	42.9	28.5	<0.001
**Specialist visits, %**			

Journal of
Clinical Medicine

≥1 visit to allergist	0.8	0.1	<0.001
≥1 visit to gastroenterologist	23.2	8.3	<0.001
≥1 visit to otolaryngologist/head and neck surgeon	12.7	4.0	<0.001
≥1 visit to pulmonologist	37.7	7.1	<0.001
≥1 visit to speech therapist	0.4	0.1	<0.001
≥1 visit to urologist	5.7	2.5	<0.001
Visited to ≥1 specialists specified above	54.2	18.2	<0.001
Visited to ≥2 different specialists specified above	21.3	3.6	<0.001
Visited to ≥3 different specialists specified above	4.7	0.4	<0.001
All missing specialty information	5.9	16.2	<0.001

Abbreviations: CC: Chronic Cough; CM: Cough Medication; SD: Standard Deviation; ED: Emergency Department; URTI: Upper Respiratory Tract Infection; URTD: Upper Respiratory Tract Disease; COPD: Chronic Obstructive Pulmonary Disease; COVID-19: Coronavirus Disease caused by the SARS-CoV-2 virus; HIV: Human Immunodeficiency Virus; RAST: Radioallergosorbent Test; GI: Gastrointestinal; CT: Computerized Tomography; MRI: Magnetic Resonance Imaging; ACE: Angiotensin Converting Enzyme; H1: Histamine-1 receptor; ICS: Inhaled Corticosteroid; LABA: Long-acting Beta-agonist; LAMA: Long-acting Muscarinic-antagonist; PDE-4: Phosphodiesterase-4; SAMA: Short-acting Muscarinic-antagonist; SABA: Short-acting Beta agonist; H2: Histamine-2 receptor; HIPAA: Health Insurance Portability and Accountability Act. * A percentage from a count of patients less than 11 or used to derive a count of patients less than 11 in other cells is not reportable according to HIPAA Privacy Rule. [a] Post-index period is 12 months after the index date. [b] Modified Elixhauser Comorbidity Index was calculated by excluding metastatic cancers, solid tumors with or without metastasis, and diseases/conditions examined individually.

Journal of Clinical Medicine

Table S7. Prescribing specialty for all post-index cough medication (CM) prescription orders by CM utilization trajectory group[a]

**In CC patients**					
	**All CC patients**	**Group 1 (No CM use)**	**Group 2 (Declining CM use)**	**Group 3 (Chronic CM use)**	**p-value**
Encounters with CM orders, N	11,801	0	9,874	1,927	
Allergist, %	0.0		*	0.0	1.000
ED physician, %	4.5		4.8	2.7	<0.001
Gastroenterologist, %	0.2		*	*	0.100
Otolaryngologist/head and neck surgeon, %	0.3		0.3	*	0.215
Primary care physician, %	58.4		57.0	65.4	<0.001
Pulmonologist, %	1.4		1.3	*	0.220
Other, %	6.7		6.6	7.2	0.327
Missing, %	28.7		29.9	22.7	<0.001
**In non-CC patients with any cough-related diagnosis**					
	**All non-CC patients**	**Group 1 (No CM use)**	**Group 2 (Declining CM use)**	**Group 3 (Chronic minimal CM use)**	**p-value**
Encounters with CM orders, N	56,839	0	39,545	17,294	
Allergist, %	0.0		*	*	0.257
ED physician, %	8.1		9.1	5.8	<0.001
Gastroenterologist, %	0.1		0.1	*	0.547
Otolaryngologist/head and neck surgeon, %	0.1		*	0.1	0.183
Primary care physician, %	54.4		56.4	50.0	<0.001
Pulmonologist, %	0.3		0.3	0.4	0.010
Other, %	4.5		4.7	4.1	0.004
Missing, %	32.4		29.4	39.4	<0.001

Abbreviations: CC: Chronic Cough; CM: Cough Medication; ED: Emergency Department; HIPAA: Health Insurance Portability and Accountability Act. * A percentage from a count of patients less than 11 or used to derive a count of patients less than 11 in other cells is not reportable according to HIPAA Privacy Rule. [a] Distinct trajectory of any CM use (i.e., opioid antitussives, benzonatate, and dextromethorphan-containing medications), excluding gabapentinoids.

Journal of
Clinical Medicine

MDPI

Table S8. Post-index characteristics of chronic cough (CC) patients by cough medication (CM) utilization trajectory group [a]

Post-index characteristics [b]	All CC patients	Group 1 (No CM use)	Group 2 (Declining CM use)	Group 3 (Chronic CM use)	p-value
N	15,566	11,222	4,105	239	
**Healthcare service use, %**					
≥1 hospitalization	41.1	41.7	39.2	46.4	0.004
No. of ED visits					<0.001
0	31.9	29.9	37.2	38.5	
1	13.7	13.8	13.6	10.9	
≥2	54.4	56.3	49.2	50.6	
No. of outpatient visits					<0.001
0	2.2	1.7	3.5	*	
1	1.3	1.4	1.2	*	
2-4	4.3	4.5	4.0	*	
≥5	92.2	92.5	91.3	92.9	
**Comorbidity index, mean (SD)**					
Elixhauser index [c]	2.7 (2.5)	2.8 (2.5)	2.4 (2.4)	3.2 (2.7)	<0.001
**Number of visits with cough-related diagnosis, mean (SD)**					
No. of visits with acute URTI	1.9 (3.8)	1.7 (3.5)	2.6 (4.4)	3.2 (4.9)	<0.001
No. of visits with bronchitis	2.5 (6.6)	2.2 (6.5)	3.1 (6.6)	5.4 (9.5)	<0.001
No. of visits with chronic URTD	1.0 (5.8)	0.9 (5.5)	1.1 (5.9)	2.5 (11.7)	<0.001
No. of visits with cough	9.1 (9.0)	8.9 (8.5)	9.9 (10.2)	9.8 (9.8)	<0.001
No. of visits with influenza	0.3 (1.6)	0.3 (1.7)	0.3 (1.2)	0.3 (1.2)	0.77
No. of visits with pneumonia	3.5 (13.7)	3.7 (14.5)	3.0 (10.0)	4.5 (23.9)	<0.001
No. of visits with any cough-related Dx	18.3 (21.1)	17.6 (21.1)	19.8 (19.8)	25.7 (32.9)	<0.001
**Respiratory comorbidities, %**					
Acute URTI	44.7	41.7	52.5	54.4	<0.001
Airway malacia	0.2	0.2	*	*	0.07
Allergic bronchopulmonary aspergillosis	0.2	0.3	*	0.0	0.51
Allergic rhinitis	28.2	26.6	32.1	37.7	<0.001
Asthma	40.8	40.1	42.0	51.5	<0.001
Bronchiectasis	5.2	5.1	5.4	8.4	0.07
Bronchiolitis obliterans	1.2	1.1	1.4	*	0.009
Bronchitis	45.7	43.2	51.6	62.3	<0.001
Chronic URTD	18.1	18.0	17.8	24.3	0.04
Chronic rhinitis	5.4	5.9	4.3	*	<0.001
Chronic sinusitis	13.9	13.4	14.7	21.3	0.001
Chronic tonsillitis	0.3	0.3	0.3	0.0	0.51
COPD	50.8	51.8	48.0	54.4	<0.001
Costochondritis	2.8	2.5	3.6	5.9	<0.001
Cough	98.2	100.0	93.7	88.3	<0.001
COVID-19/coronavirus	7.7	8.3	6.4	6.7	<0.001
Cystic fibrosis	1.3	1.5	0.9	*	0.02
Eosinophilic bronchitis/pneumonia	0.7	0.7	0.6	*	0.74
Hemoptysis	5.9	5.8	6.0	9.2	0.07
Hyperventilation	0.3	0.3	0.3	0.0	0.57
Influenza	6.8	6.4	7.9	9.6	0.001
Nasal polyp disease	0.8	0.9	0.7	*	0.40
Obstructive sleep apnea	18.4	17.4	20.4	28.0	<0.001

Journal of
Clinical Medicine

Pertussis	8.8	9.0	8.0	10.9	0.09
Pneumonia	30.6	31.5	28.1	32.2	<0.001
Pulmonary fibrosis	7.3	6.9	8.3	12.1	<0.001
Respiratory foreign body	2.4	2.6	2.0	*	0.10
Sarcoidosis	1.2	1.0	1.4	*	0.10
SARS	0.1	0.2	*	0.0	0.35
Tonsillar enlargement	0.5	0.5	0.6	*	0.21
Upper airway cough syndrome	6.3	5.5	8.3	8.4	<0.001
Vocal cord dysfunction	1.2	1.1	1.4	*	0.02
**Non-respiratory comorbidities, %**					
Alcohol use disorder	6.0	6.0	5.8	9.2	0.09
Anxiety disorders	39.5	39.9	37.9	49.8	<0.001
Atrial fibrillation	11.6	11.7	11.2	14.6	0.24
Coronary artery disease	39.6	39.1	40.4	49.0	0.004
Eosinophilia	0.9	0.9	1.1	0.0	0.14
Eosinophilic esophagitis/gastroenteritis	0.2	0.3	*	*	0.19
Gastroesophageal reflux disease	48.8	47.7	51.0	61.1	<0.001
Granulomatosis	0.2	0.2	*	0.0	0.72
Heart failure	21.5	21.8	20.3	25.9	0.04
HIV infection	3.6	4.0	2.7	*	<0.001
Hypertension	68.0	68.0	67.3	79.9	<0.001
Immune deficiency	2.3	2.1	3.1	*	0.001
Immunosuppression treatments	*	*	*	*	0.07
Mast cell disease	1.0	1.0	0.9	*	0.15
Mood disorders	39.8	41.6	34.9	38.5	<0.001
Musculoskeletal disorders	75.7	76.0	74.5	83.3	0.004
Non-opioid substance use disorders	14.9	15.4	13.4	17.6	0.003
Obesity	36.5	34.7	40.4	51.1	<0.001
Opioid use disorder	5.5	5.8	4.5	6.3	0.006
Other immune disorders	8.7	9.0	7.9	11.3	0.04
Peripheral vascular disease	9.5	10.0	8.2	8.4	0.005
Relapsing polychondritis	0.3	0.2	0.4	*	0.009
Rib fracture	1.3	1.3	1.3	*	0.08
Sleep disturbance	15.2	14.5	16.3	27.2	<0.001
Stress incontinence	4.1	4.0	4.3	5.0	0.48
Subconjunctival hemorrhage	0.9	1.0	0.7	*	0.33
Transplantation of major organ	1.7	1.4	2.3	*	<0.001
Vomiting	10.9	11.5	9.2	12.1	<0.001
**Procedures, %**					
Allergy RAST testing	5.3	5.9	3.7	*	<0.001
Barium swallow or upper GI imaging	8.3	8.5	7.7	8.8	0.28
Bronchoscopy	5.9	5.8	6.1	7.1	0.56
Chest CT/MRI/ultrasound	35.2	35.9	33.5	32.2	0.01
Chest X-ray	74.6	78.2	65.9	59.0	<0.001
Complete blood cell count	68.5	73.1	56.9	51.1	<0.001
Esophageal endoscopy	11.9	12.6	9.9	11.7	<0.001
Esophageal pH studies/manometry	1.4	1.3	1.6	*	0.04
Laryngoscopy	6.9	6.7	7.4	6.3	0.04
Methacholine challenge	0.2	0.3	*	*	0.25
Nasal/sinus endoscopy	3.8	3.7	3.8	5.4	0.60
Sinus X-ray/CT	7.5	7.9	6.3	7.1	0.004

Journal of Clinical Medicine

Spirometry	24.6	25.8	21.5	21.3	0.38
**Cough medications**					
Benzonatate, %	19.7	*	70.2	72.4	<0.001
Dextromethorphan, %	7.7	*	26.7	42.7	<0.001
Gabapentinoids, %	10.3	5.6	21.9	29.7	<0.001
Opioid antitussives, %	11.2	*	38.6	64.4	<0.001
No. of any CM prescriptions including gabapentinoids, mean (SD)	1.1 (2.3)	0.2 (0.9)	3.1 (2.5)	9.6 (4.9)	<0.001
No. of any CM prescriptions excluding gabapentinoids, mean (SD)	0.8 (1.6)	0.0 (0.0)	2.4 (1.5)	8.1 (3.0)	<0.001
**Cardiovascular medications, %**					
ACE inhibitors	8.9	4.9	18.9	26.8	<0.001
**Respiratory medications (oral, inhaled, or nasal), %**					
Biologics for asthma	0.3	0.2	0.5	*	<0.001
H1 antihistamines	20.6	10.7	45.4	62.3	<0.001
ICS monotherapy	5.7	3.0	12.1	18.0	<0.001
ICS/LABA combination	13.5	6.9	29.6	46.9	<0.001
ICS/LABA/LAMA combination	0.2	0.1	0.4	0.0	0.001
LABA monotherapy	0.8	0.2	2.0	*	<0.001
LABA/LAMA combination	0.3	0.2	0.6	*	<0.001
LAMA monotherapy	4.6	2.2	10.5	20.1	<0.001
Leukotriene modifiers	10.0	4.8	22.8	35.2	<0.001
Mast cell inhibitors inhaled	0.0	0.0	0.0	0.0	N/A
Mast cell inhibitors nasal	*	0.0	*	*	0.004
Mast cell inhibitors oral	*	*	*	0.0	0.48
Nasal antihistamines	1.9	0.8	4.8	4.6	<0.001
Nasal antihistamines/corticosteroids	0.2	0.1	0.6	0.0	<0.001
Nasal corticosteroids	15.3	7.1	36.0	46.4	<0.001
Nasal SAMA	1.0	0.5	2.2	*	<0.001
PDE4 inhibitors for COPD	0.3	0.1	0.7	*	<0.001
SABA singly inhaled	26.1	13.1	59.1	69.5	<0.001
SABA/SAMA combination	12.3	5.5	28.8	45.2	<0.001
SAMA singly inhaled	5.4	2.5	12.1	25.9	<0.001
Theophylline	0.4	0.1	1.0	*	<0.001
**Gastrointestinal medications (oral), %**					
H2 antihistamines	10.6	6.1	21.6	31.0	<0.001
Proton pump inhibitors	22.1	12.4	46.2	62.3	<0.001
**Miscellaneous medications (oral), %**					
Antibiotics for respiratory conditions	35.9	18.2	81.0	90.8	<0.001
Corticosteroids	20.8	9.0	51.1	59.4	<0.001
**Pain medications, psychotherapeutics, others (oral), %**					
Antidepressants	17.3	9.5	36.9	45.6	<0.001
Antipsychotics	6.1	4.1	11.5	12.1	<0.001
Benzodiazepines	14.4	8.6	28.0	49.4	<0.001
Muscle relaxants	10.7	5.0	24.5	40.2	<0.001
Non-benzodiazepine hypnotics	3.1	1.5	6.7	16.3	<0.001
Opioid analgesics	22.2	12.2	47.0	64.9	<0.001
Other anxiolytics	1.8	0.9	3.9	5.0	<0.001
Other neuromodulators	7.4	4.8	13.9	18.4	<0.001
**Polypharmacy, %**					
≥3 different medications	42.9	26.9	83.6	93.3	<0.001

**Specialist visits, %**					
≥1 visit to allergist	0.8	0.6	1.4	0.0	<0.001
≥1 visit to gastroenterologist	23.2	24.4	20.0	18.8	<0.001
≥1 visit to otolaryngologist/head and neck surgeon	12.7	13.1	12.0	9.6	0.08
≥1 visit to pulmonologist	37.7	40.1	31.4	36.4	<0.001
≥1 visit to speech therapist	0.4	0.5	0.3	0.0	0.23
≥1 visit to urologist	5.7	6.0	4.8	5.4	0.02
Visited to ≥1 specialists specified above	54.2	57.2	46.5	49.4	<0.001
Visited to ≥2 different specialists specified above	21.3	22.4	18.4	18.4	<0.001
Visited to ≥3 different specialists specified above	4.7	4.8	4.5	*	0.13
All missing specialty information	5.9	3.5	12.0	11.3	<0.001

Abbreviations: CC: Chronic Cough; CM: Cough Medication; SD: Standard Deviation; URTI: Upper Respiratory Tract Infection; URTD: Upper Respiratory Tract Disease; Dx: Diagnosis; COPD: Chronic Obstructive Pulmonary Disease; COVID-19: Coronavirus Disease caused by the SARS-CoV-2 virus; HIV: Human Immunodeficiency Virus; RAST: Radioallergosorbent Test; GI: Gastrointestinal; CT: Computerized Tomography; MRI: Magnetic Resonance Imaging; ACE: Angiotensin Converting Enzyme; H1: Histamine-1 receptor; ICS: Inhaled Corticosteroid; LABA: Long-acting Beta-agonist; LAMA: Long-acting Muscarinic-antagonist; PDE-4: Phosphodiesterase-4; SAMA: Short-acting Muscarinic-antagonist; SABA: Short-acting Beta agonist; H2: Histamine-2 receptor; HIPAA: Health Insurance Portability and Accountability Act. * A percentage from a count of patients less than 11 or used to derive a count of patients less than 11 in other cells is not reportable according to HIPAA Privacy Rule. [a] Distinct trajectory of any CM use (i.e., opioid antitussives, benzonatate, and dextromethorphan-containing medications), excluding gabapentinoids. [b] Post-index period is 12 months after the index date. [c] Modified Elixhauser Comorbidity Index was calculated by excluding metastatic cancers, solid tumors with or with-out metastasis, and diseases/conditions examined individually.

Journal of
Clinical Medicine

MDPI

Table S9. Post-index characteristics of non-chronic cough (CC) patients with any cough-related diagnosis by cough medication (CM) utilization trajectory group [a]

Post-index characteristics [b]	All non-CC patients	Group 1 (No CM use)	Group 2 (Declining CM use)	Group 3 (Chronic minimal CM use)	p-value
N	655,260	611,496	31,805	11,959	
**Healthcare service use, %**					
≥1 hospitalization	28.1	28.5	22.7	25.6	<0.001
No. of ED visits					<0.001
0	41.8	41.4	46.4	49.4	
1	19.9	20.2	18.2	12.4	
≥2	38.2	38.4	35.4	38.2	
No. of outpatient visits					<0.001
0	9.2	9.0	14.8	8.9	
1	6.7	6.7	7.6	3.4	
2-4	22.0	22.2	21.0	14.8	
≥5	62.1	62.1	56.6	73.0	
**Comorbidity index, mean (SD)**					
Elixhauser index [c]	1.3 (1.8)	1.3 (1.8)	1.1 (1.6)	1.3 (0.9)	<0.001
**Number of visits with cough-related diagnosis, mean (SD)**					
No. of visits with acute URTI	1.2 (2.0)	1.2 (2.0)	1.5 (2.2)	2.4 (3.1)	<0.001
No. of visits with bronchitis	0.6 (1.8)	0.5 (1.8)	1.0 (1.9)	1.4 (2.7)	<0.001
No. of visits with chronic URTD	0.4 (2.1)	0.4 (2.1)	0.2 (1.8)	0.5 (2.3)	<0.001
No. of visits with cough	0.9 (1.8)	0.8 (1.7)	1.7 (2.7)	2.1 (3.1)	<0.001
No. of visits with influenza	0.1 (0.9)	0.1 (0.8)	0.2 (1.0)	0.3 (1.9)	<0.001
No. of visits with pneumonia	0.7 (3.9)	0.7 (4.0)	0.5 (2.0)	0.8 (3.6)	<0.001
No. of visits with any cough-related dx	3.8 (5.7)	3.7 (5.6)	5.0 (5.1)	7.4 (7.5)	<0.001
**Respiratory comorbidities, %**					
Acute URTI	49.4	49.0	51.0	66.8	<0.001
Airway malacia	1.4	1.3	2.0	3.9	<0.001
Allergic bronchopulmonary aspergillosis	0.0	0.0	*	*	<0.001
Allergic rhinitis	9.5	9.3	10.6	16.5	<0.001
Asthma	13.3	13.2	14.3	20.7	<0.001
Bronchiectasis	0.5	0.5	0.5	1.2	<0.001
Bronchiolitis obliterans	0.2	0.2	0.2	0.4	<0.001
Bronchitis	22.4	21.4	35.6	39.7	<0.001
Chronic URTD	12.8	13.1	6.3	14.9	<0.001
Chronic rhinitis	2.5	2.6	0.8	2.6	<0.001
Chronic sinusitis	9.8	10.0	5.5	11.1	<0.001
Chronic tonsillitis	0.2	0.2	0.1	0.2	<0.001
COPD	18.3	18.0	19.8	27.0	<0.001
Costochondritis	0.9	0.8	1.2	1.4	<0.001
Cough	39.2	37.9	56.5	58.8	<0.001
COVID-19/coronavirus	3.3	3.2	3.3	4.0	<0.001
Cystic fibrosis	0.2	0.2	0.1	0.2	0.51
Eosinophilic bronchitis/pneumonia	0.1	0.1	0.1	0.1	0.008
Hemoptysis	1.6	1.6	1.6	2.4	<0.001
Hyperventilation	0.1	0.1	0.1	0.1	0.14
Influenza	6.1	5.9	9.0	9.8	<0.001
Nasal polyp disease	0.4	0.4	0.1	0.4	<0.001

Journal of
Clinical Medicine

Obstructive sleep apnea	6.1	6.0	6.9	9.4	<0.001
Pertussis	6.5	6.7	4.4	6.6	<0.001
Pneumonia	14.6	14.7	13.0	16.3	<0.001
Pulmonary fibrosis	1.3	1.3	1.3	2.5	<0.001
Respiratory foreign body	0.5	0.5	0.3	0.5	<0.001
Sarcoidosis	0.3	0.3	0.3	0.5	<0.001
SARS	0.1	0.1	*	0.0	<0.001
Tonsillar enlargement	0.4	0.4	0.2	0.5	<0.001
Upper airway cough syndrome	1.4	1.3	2.0	3.9	<0.001
Vocal cord dysfunction	0.2	0.2	0.1	0.3	0.003
**Non-respiratory comorbidities, %**					
Alcohol use disorder	3.7	3.7	2.8	3.6	<0.001
Anxiety disorder	20.0	20.0	18.7	23.3	<0.001
Atrial fibrillation	6.0	6.0	5.5	6.8	<0.001
Coronary artery disease	24.3	24.3	22.2	27.2	<0.001
Eosinophilia	0.2	0.2	0.1	0.3	0.002
Eosinophilic esophagitis/gastroenteritis	0.1	0.1	0.1	0.1	0.06
Gastroesophageal reflux disease	18.3	18.1	18.7	24.7	<0.001
Granulomatosis	0.1	0.1	*	*	0.14
Heart failure	8.7	8.8	6.9	10.2	<0.001
HIV infection	1.6	1.6	0.8	1.2	<0.001
Hypertension	40.7	40.4	43.6	46.3	<0.001
Immune deficiency	0.7	0.7	0.4	0.8	<0.001
Immunosuppression treatments	0.0	0.0	*	0.0	0.84
Mast cell disease	0.3	0.3	0.2	0.4	0.001
Mood disorder	20.2	20.4	16.0	20.3	<0.001
Musculoskeletal disorders	48.4	48.3	46.5	54.1	<0.001
Non-opioid substance use disorders	8.6	8.7	6.0	7.9	<0.001
Obesity	20.9	20.6	24.2	27.0	<0.001
Opioid use disorder	2.4	2.4	1.4	2.0	<0.001
Other immune disorders	4.1	4.2	3.3	5.0	<0.001
Peripheral vascular disease	3.9	4.0	2.5	3.7	<0.001
Relapsing polychondritis	0.2	0.2	0.1	0.2	0.17
Rib fracture	0.6	0.6	0.5	0.7	0.02
Sleep disturbance	5.7	5.6	6.3	8.7	<0.001
Stress incontinence	1.4	1.3	1.4	2.1	<0.001
Subconjunctival hemorrhage	0.4	0.4	0.3	0.6	0.006
Transplantation of major organ	0.6	0.6	0.4	0.6	<0.001
Vomiting	4.2	4.2	3.9	4.7	<0.001
**Procedures, %**					
Allergy RAST testing	0.7	0.8	0.3	0.8	<0.001
Barium swallow or upper GI imaging	1.9	1.9	1.2	2.1	<0.001
Bronchoscopy	1.1	1.1	0.7	1.6	<0.001
Chest CT/MRI/ultrasound	9.5	9.5	7.7	12.6	<0.001
Chest X-ray	39.7	40.0	34.1	40.5	<0.001
Complete blood cell count	47.3	48.4	30.2	37.6	<0.001
Esophageal endoscopy	4.4	4.5	3.0	4.4	<0.001
Esophageal pH studies/manometry	0.2	0.2	0.2	0.2	<0.001
Laryngoscopy	1.3	1.3	0.7	1.9	0.37
Methacholine challenge	0.0	0.0	*	*	<0.001
Nasal/sinus endoscopy	1.6	1.7	0.5	1.8	0.86
Sinus X-ray/CT	2.9	2.9	1.2	2.5	<0.001

Journal of Clinical Medicine

Spirometry	3.4	3.4	2.0	4.9	<0.001
**Cough medications**					
Benzonatate, %	4.4	0.1	63.8	65.9	<0.001
Dextromethorphan, %	1.2	0.0	16.2	21.0	<0.001
Gabapentinoids, %	3.9	3.6	7.3	10.6	<0.001
Opioid antitussives, %	2.1	0.0	30.8	29.7	<0.001
No. of any CM prescriptions including gabapentinoids, mean (SD)	0.2 (0.7)	0.1 (0.6)	1.4 (1.0)	1.7 (1.5)	<0.001
No. of any CM prescriptions excluding gabapentinoids, mean (SD)	0.1 (0.4)	0.0 (0.0)	1.2 (0.6)	1.5 (0.9)	<0.001
**Cardiovascular medications, %**					
ACE inhibitors	5.4	4.8	12.5	14.9	<0.001
**Respiratory medications (oral, inhaled, or nasal), %**					
Biologics for asthma	0.0	0.0	*	0.1	<0.001
H1 antihistamines	9.0	8.1	19.7	28.7	<0.001
ICS monotherapy	0.8	0.7	2.2	4.2	<0.001
ICS/LABA combination	2.3	1.9	5.5	11.5	<0.001
ICS/LABA/LAMA combination	0.0	0.0	*	0.1	<0.001
LABA monotherapy	0.1	0.1	0.2	0.4	<0.001
LABA/LAMA combination	0.1	0.0	0.1	0.2	<0.001
LAMA monotherapy	0.7	0.6	1.3	3.0	<0.001
Leukotriene modifiers	1.7	1.4	4.4	8.1	<0.001
Mast cell inhibitors inhaled	*	*	0.0	0.0	0.49
Mast cell inhibitors nasal	0.0	0.0	*	*	0.008
Mast cell inhibitors oral	0.0	0.0	0.0	0.0	0.74
Nasal antihistamines	0.5	0.4	0.9	2.3	<0.001
Nasal antihistamines/corticosteroids	0.1	0.0	0.1	0.3	<0.001
Nasal corticosteroids	5.2	4.2	15.7	26.6	<0.001
Nasal SAMA	0.2	0.1	0.5	0.8	<0.001
PDE4 inhibitors for COPD	0.0	0.0	0.0	0.2	<0.001
SABA singly inhaled	7.5	5.8	28.3	37.3	<0.001
SABA/SAMA combination	3.4	2.9	10.4	15.2	<0.001
SAMA singly inhaled	0.9	0.7	2.3	4.4	<0.001
Theophylline	0.0	0.0	0.1	0.3	<0.001
**Gastrointestinal medications (oral), %**					
H2 antihistamines	4.6	4.3	8.6	11.8	<0.001
Proton pump inhibitors	7.7	7.0	15.6	23.5	<0.001
**Miscellaneous medications (oral), %**					
Antibiotics for respiratory conditions	24.6	21.7	63.4	74.7	<0.001
Corticosteroids	7.2	5.8	25.0	33.5	<0.001
**Pain medications, psychotherapeutics, others (oral), %**					
Antidepressants	7.8	7.1	16.7	22.5	<0.001
Antipsychotics	2.6	2.5	4.1	5.8	<0.001
Benzodiazepines	7.7	7.2	12.9	19.2	<0.001
Muscle relaxants	4.9	4.4	11.6	15.3	<0.001
Non-benzodiazepine hypnotics	1.5	1.3	3.3	4.9	<0.001
Opioid analgesics	13.7	12.6	26.8	32.7	<0.001
Other anxiolytics	0.6	0.5	1.4	1.9	<0.001
Other neuromodulators	3.5	3.2	6.1	8.9	<0.001
**Polypharmacy, %**					
≥3 different medications	28.5	26.3	54.3	72.8	<0.001

Journal of
Clinical Medicine

**Specialist visits, %**					
≥1 visit to allergist	0.1	0.1	0.2	0.3	<0.001
≥1 visit to gastroenterologist	8.3	8.4	6.8	8.2	<0.001
≥1 visit to otolaryngologist/head and neck surgeon	4.0	4.1	2.3	4.0	<0.001
≥1 visit to pulmonologist	7.1	7.3	4.0	7.6	<0.001
≥1 visit to speech therapist	0.1	0.1	*	*	<0.001
≥1 visit to urologist	2.5	2.6	2.0	2.5	<0.001
Visited to ≥1 specialists specified above	18.2	18.5	13.0	18.1	<0.001
Visited to ≥2 different specialists specified above	3.6	3.6	2.0	4.1	<0.001
Visited to ≥3 different specialists specified above	0.4	0.5	0.2	0.6	<0.001
All missing specialty information	16.2	16.0	17.4	25.3	<0.001

Abbreviations: CC: Chronic Cough; CM: Cough Medication; SD: Standard Deviation; URTI: Upper Respiratory Tract Infection; URTD: Upper Respiratory Tract Disease; Dx: Diagnosis; COPD: Chronic Obstructive Pulmonary Disease; COVID-19: Coronavirus Disease caused by the SARS-CoV-2 virus; HIV: Human Immunodeficiency Virus; RAST: Radioallergosorbent Test; GI: Gastrointestinal; CT: Computerized Tomography; MRI: Magnetic Resonance Imaging; ACE: Angiotensin Converting Enzyme; H1: Histamine-1 receptor; ICS: Inhaled Corticosteroid; LABA: Long-acting Beta-agonist; LAMA: Long-acting Muscarinic-antagonist; PDE-4: Phosphodiesterase-4; SAMA: Short-acting Muscarinic-antagonist; SABA: Short-acting Beta agonist; H2: Histamine-2 receptor; HIPAA: Health Insurance Portability and Accountability Act. * A percentage from a count of patients less than 11 or used to derive a count of patients less than 11 in other cells is not reportable according to HIPAA Privacy Rule. [a] Distinct trajectory of any CM use (i.e., opioid antitussives, benzonatate, and dextromethorphan-containing medications), excluding gabapentinoids. [b] Post-index period is 12 months after the index date. [c] Modified Elixhauser Comorbidity Index was calculated by excluding metastatic cancers, solid tumors with or with-out metastasis, and diseases/conditions examined individually.

Journal of
Clinical Medicine

MDPI

Table S10. Odds ratios for pre-index factors associated with cough medication (CM) utilization trajectories[a] in chronic cough (CC) patients

	Group 2 (Declining CM use)† OR [95% CI]	Group 3 (Chronic CM use)† OR [95% CI]
**Socio-demographics**		
Age‡	1.00 [1.00, 1.00]	1.02 [1.01, 1.03]
Sex		
Male	Reference	Reference
Female	1.21 [1.10, 1.33]	1.00 [0.74, 1.36]
Race		
White	Reference	Reference
Black	1.15 [1.04, 1.27]	1.78 [1.30, 2.44]
Other	1.11 [0.94, 1.30]	1.47 [0.84, 2.58]
Ethnicity		
Non-Hispanic/unknown	Reference	Reference
Hispanic	0.77 [0.68, 0.87]	0.81 [0.52, 1.27]
Payer		
Medicare	Reference	Reference
Medicaid	0.67 [0.60, 0.76]	0.53 [0.34, 0.81]
Commercial	3.03 [2.61, 3.52]	4.00 [2.67, 5.99]
Other	1.14 [0.94, 1.39]	1.05 [0.53, 2.10]
**Healthcare service use**		
No. of outpatient visits		
0	Reference	Reference
1	0.98 [0.80, 1.20]	0.74 [0.36, 1.51]
2-4	0.87 [0.73, 1.04]	0.53 [0.29, 0.97]
≥5	0.70 [0.59, 0.83]	0.64 [0.38, 1.08]
**Respiratory comorbidities**		
Asthma	1.13 [1.01, 1.27]	1.74 [1.26, 2.39]
Chronic URTD	0.77 [0.66, 0.91]	1.15 [0.78, 1.71]
Cough	0.71 [0.64, 0.79]	0.87 [0.63, 1.19]
Cystic fibrosis	0.18 [0.11, 0.32]	0.00 [0.00, 99.00]
Obstructive sleep apnea	1.28 [1.10, 1.49]	1.10 [0.72, 1.70]
Pulmonary fibrosis	1.35 [1.05, 1.73]	2.03 [1.11, 3.72]
**Non-respiratory comorbidities**		
Hypertension	0.95 [0.86, 1.05]	0.75 [0.54, 1.04]
Immune deficiency	0.43 [0.27, 0.67]	1.26 [0.52, 3.06]
Musculoskeletal disorders	0.85 [0.77, 0.94]	0.64 [0.46, 0.90]
Obesity	1.24 [1.11, 1.38]	1.60 [1.16, 2.22]
Sleep disturbance	0.82 [0.69, 0.97]	1.45 [0.97, 2.17]
**Procedures**		
Complete blood cell count	0.60 [0.54, 0.66]	0.38 [0.27, 0.55]
Chest X-ray	0.68 [0.61, 0.76]	0.57 [0.40, 0.82]
Spirometry	0.81 [0.67, 0.99]	0.35 [0.16, 0.75]
**Concomitant medication use**		
Antibiotics for respiratory conditions	2.37 [2.11, 2.65]	2.93 [2.12, 4.05]
Antidepressants	1.56 [1.36, 1.79]	1.30 [0.92, 1.83]
Benzodiazepines	1.09 [0.94, 1.27]	1.93 [1.38, 2.71]
H_1 antihistamines	1.36 [1.18, 1.58]	1.49 [1.05, 2.09]

Journal of
Clinical Medicine

Muscle relaxants	1.41 [1.18, 1.68]	2.34 [1.63, 3.37]
Nasal corticosteroids	1.55 [1.31, 1.84]	1.87 [1.29, 2.71]
Opioid analgesics	1.75 [1.53, 2.00]	3.24 [2.29, 4.57]
Oral corticosteroids	1.98 [1.70, 2.31]	2.36 [1.66, 3.34]
**Polypharmacy**		
≥3 different medications	2.71 [2.43, 3.03]	3.47 [2.38, 5.07]

Abbreviations: CC: Chronic Cough; CM: Cough Medication; URTD: Upper Respiratory Tract Disease; H_1: Histamine-1 receptor. † It was compared to Group 1 in a multinomial logistic regression. ‡ Continuous variables. [a] Distinct trajectory of any CM use (i.e., opioid antitussives, benzonatate, and dextromethorphan-containing medications), excluding gabapentinoids.

Journal of
Clinical Medicine

Figure S1. Chronic cough identification algorithm

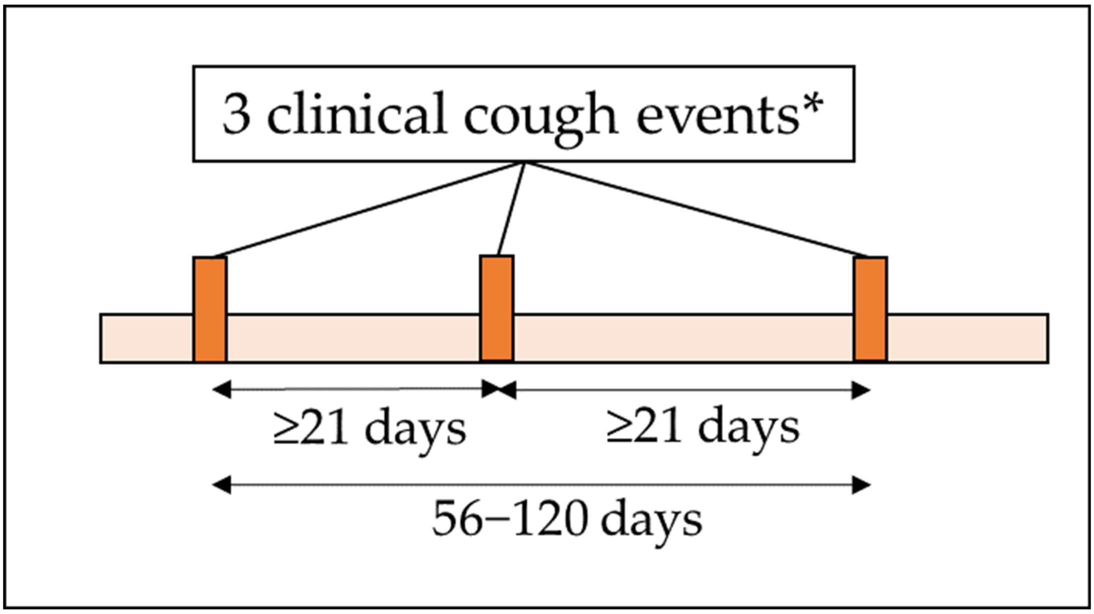

* A diagnosis of cough (ICD-9-CM: 786.2 or ICD-10-CM: R05) or a CM prescription order, including opioid antitussives, benzonatate, or dextromethorphan-containing medications

Journal of
Clinical Medicine

Figure S2. Study design diagram for chronic cough (CC) patients in group-based trajectory modeling (GBTM) analysis

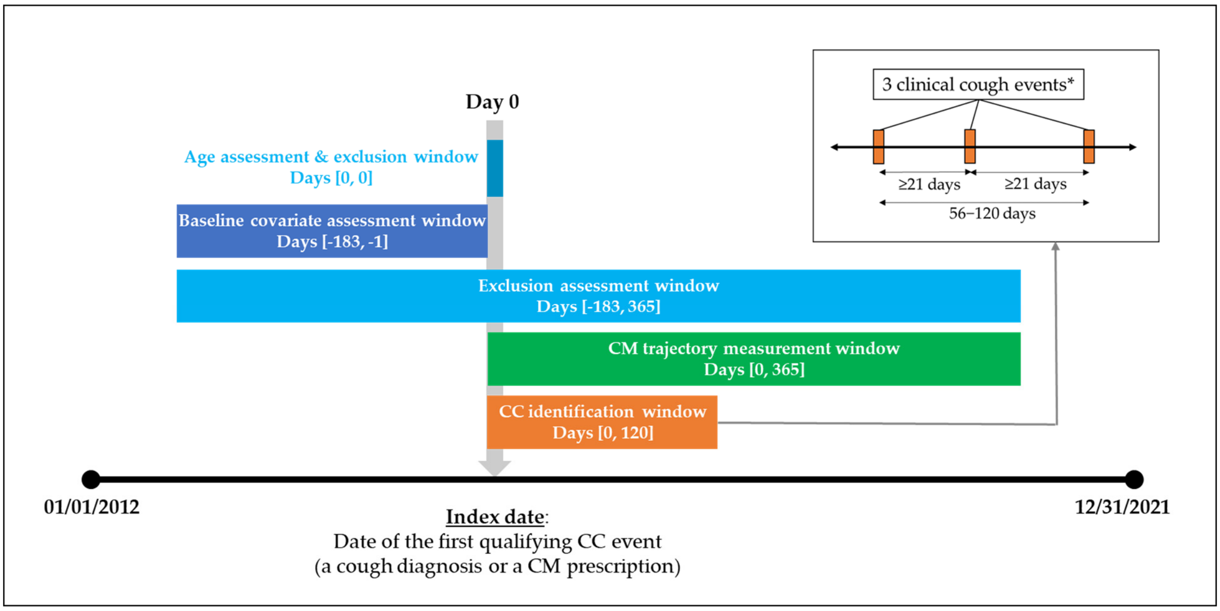

Abbreviations: CC: Chronic Cough; CM: Cough Medication. * A diagnosis of cough (ICD-9-CM: 786.2 or ICD-10-CM: R05) or a CM prescription order, including opioid antitussives, benzonatate, or dextromethorphan-containing medications

Journal of
Clinical Medicine

Figure S3. Study design diagram for non-chronic cough (CC) patients with any cough-related diagnosis in group-based trajectory modeling (GBTM) analysis

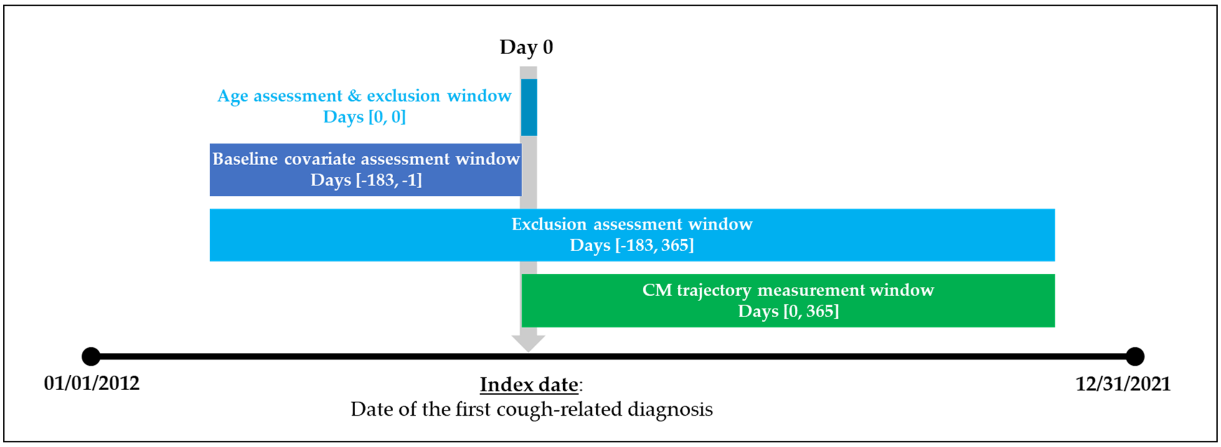

Abbreviations: CM: Cough Medication.

Journal of
Clinical Medicine
MDPI

Figure S4. Flowchart for group-based trajectory modeling (GBTM) analysis: 2012–2021 OneFlorida data

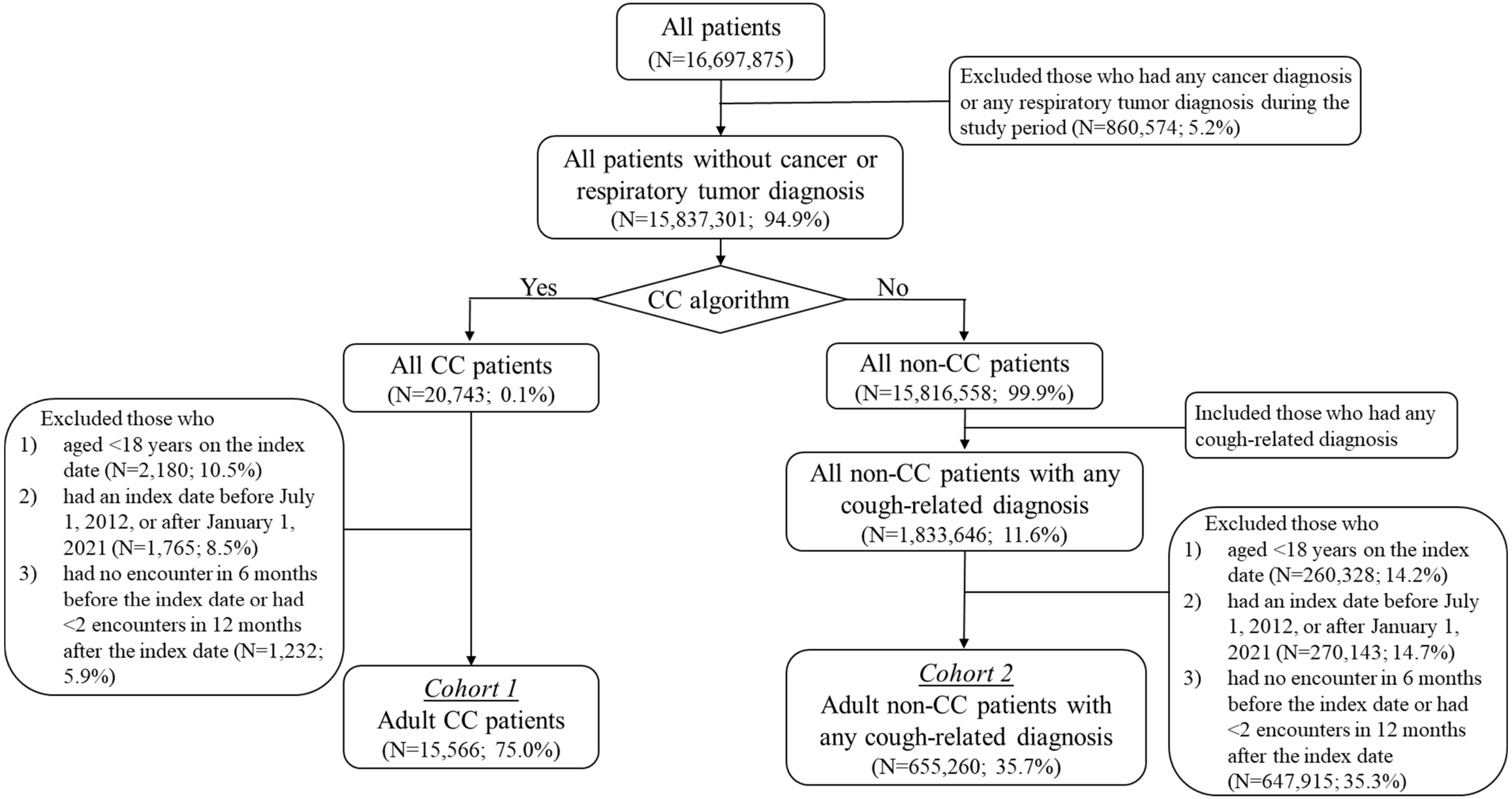

Abbreviations: CC: Chronic Cough.

Journal of
Clinical Medicine

Figure S5. Top 10 post-index respiratory comorbidities: 2012–2021 OneFlorida data

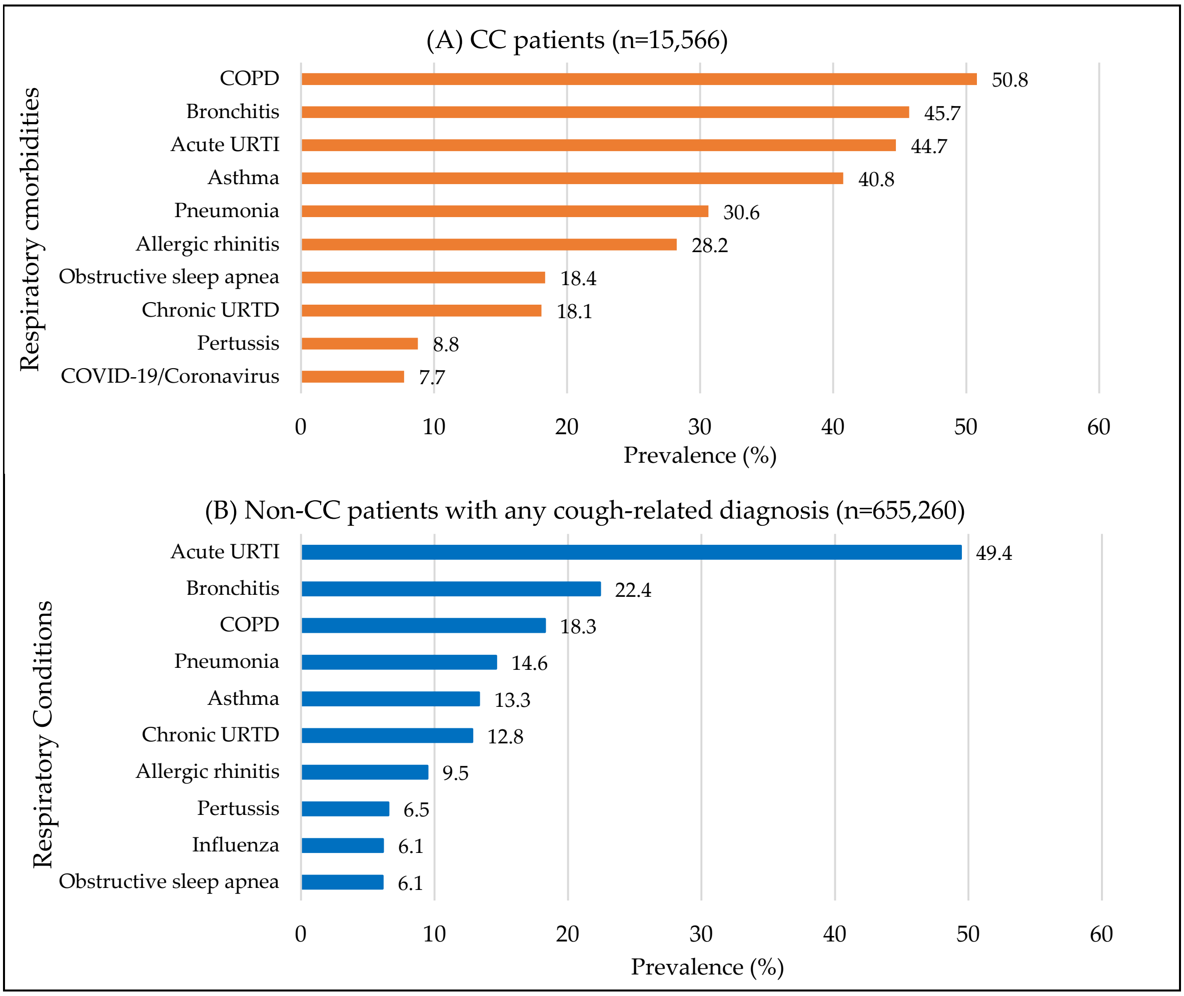

Abbreviations: CC: Chronic Cough; COPD: Chronic Obstructive Pulmonary Disease; URTI: Upper Respiratory Tract Infection; URTD: Upper Respiratory Tract Disease; COVID-19: Coronavirus Disease caused by the SARS-CoV-2 virus.

Journal of
Clinical Medicine
MDPI

Figure S6. Top 10 post-index non-respiratory comorbidities: 2012–2021 OneFlorida data

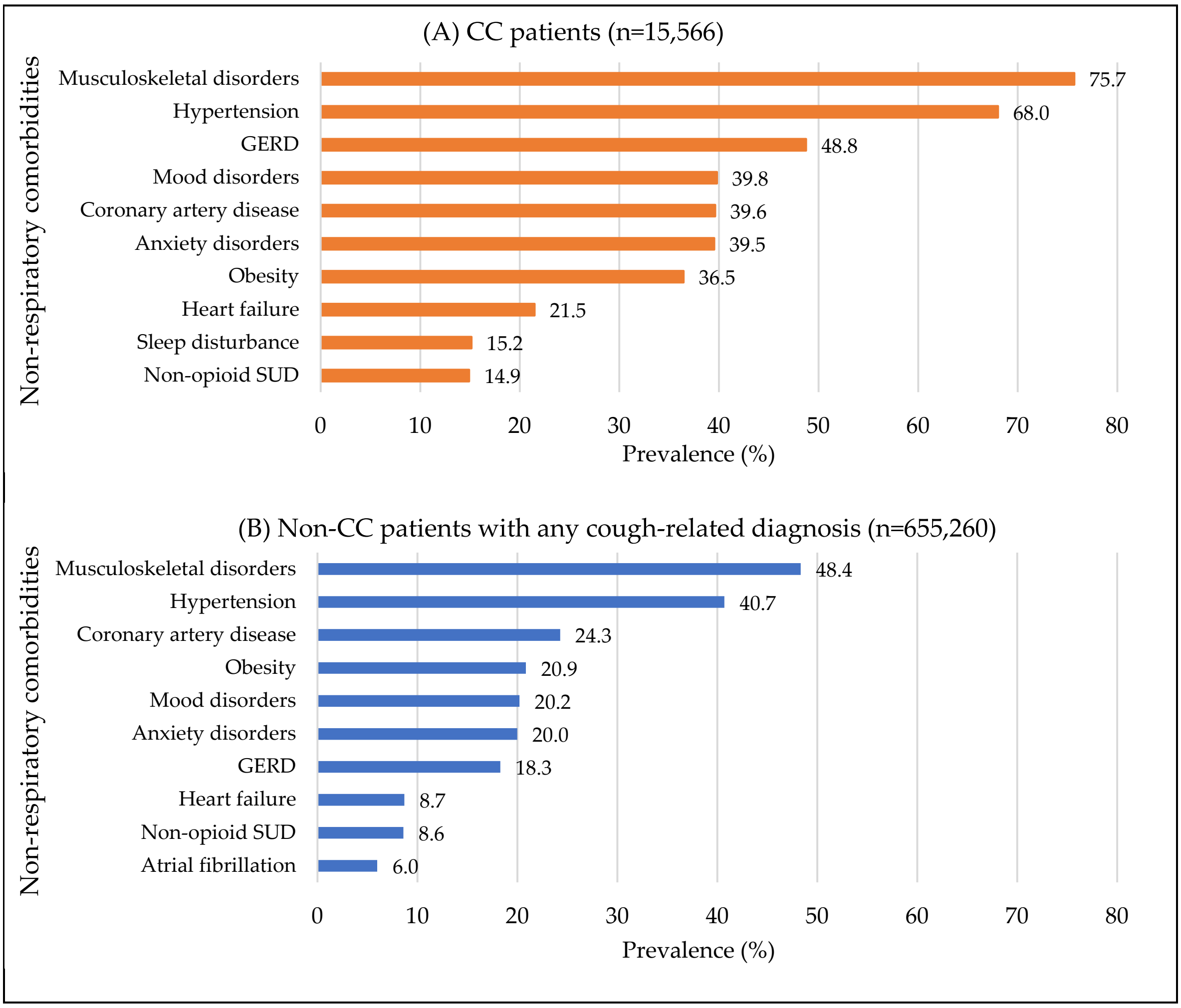

Abbreviations: CC: Chronic Cough; GERD: Gastroesophageal Reflux Disease; SUD: Substance Use Disorder.

Journal of
Clinical Medicine

Figure S7. Top 10 post-index concomitant medication use: 2012–2021 OneFlorida data

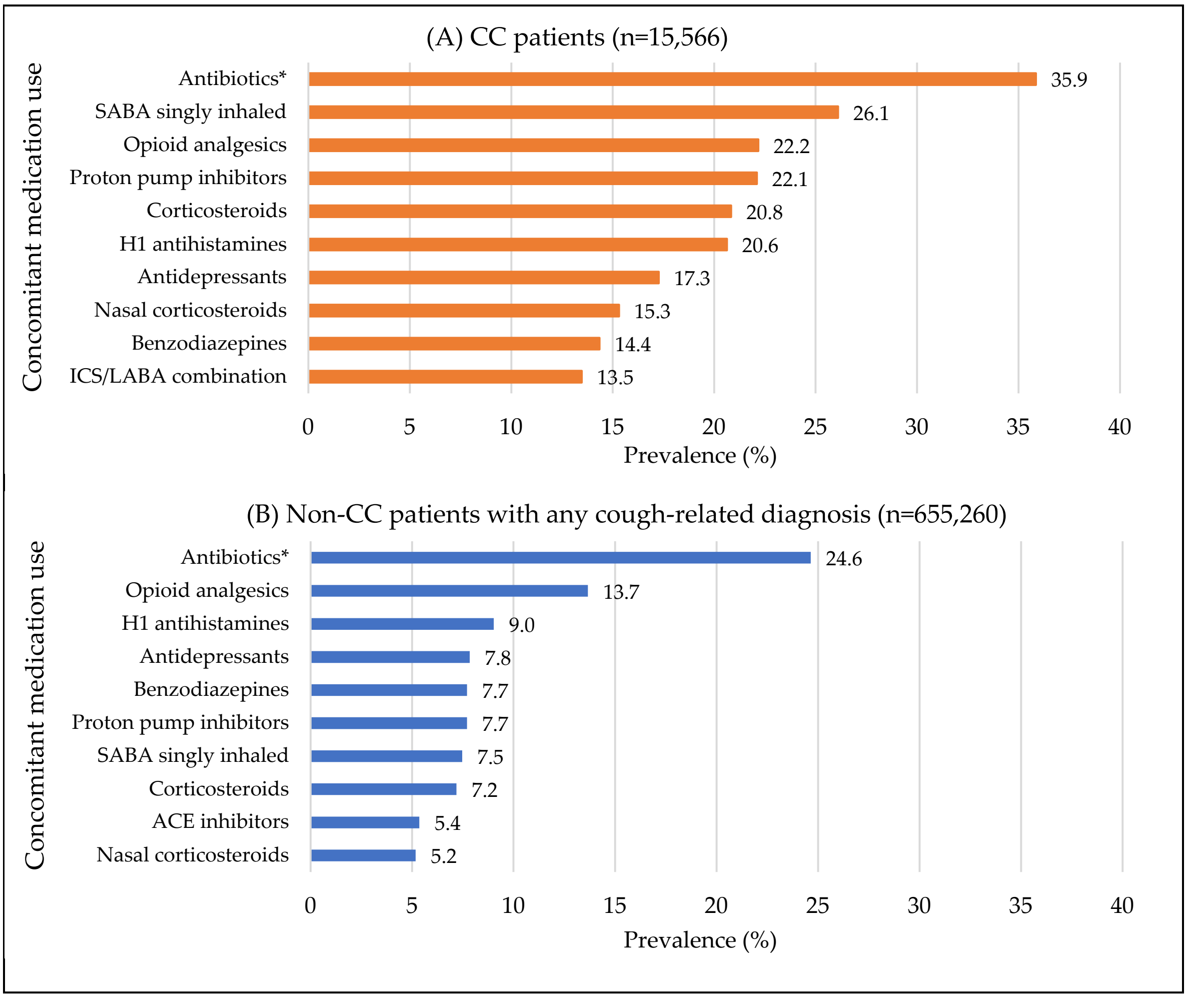

Abbreviations: CC: Chronic Cough; SABA: Short-acting Beta-agonist; H1: Histamine-1 receptor; ICS: Inhaled Corticosteroid; LABA: Long-acting Beta-agonist; ACE: Angiotensin Converting Enzyme. * Antibiotics for respiratory conditions.

Journal of
Clinical Medicine

MDPI

Figure S8. Post-index cough medication (CM) use: 2012–2021 OneFlorida data

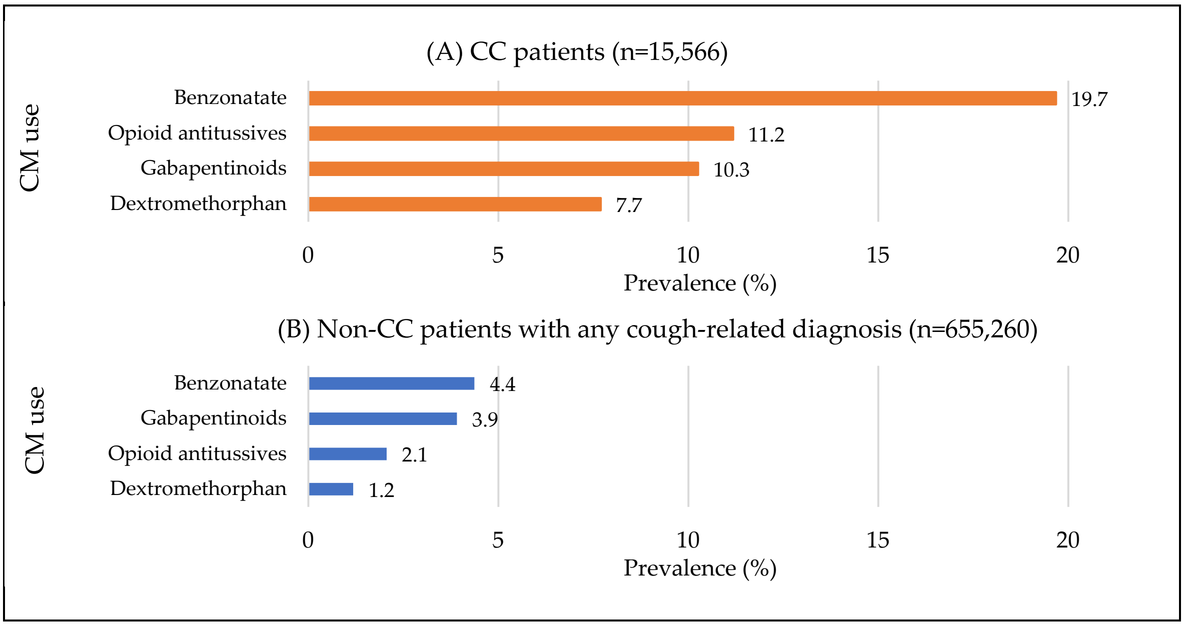

Abbreviations: CM: Cough Medication; CC: Chronic Cough.

Journal of
Clinical Medicine

Figure S9. Forest plot showing odds ratios for factors associated with cough medication (CM) utilization trajectories in chronic cough (CC) patients

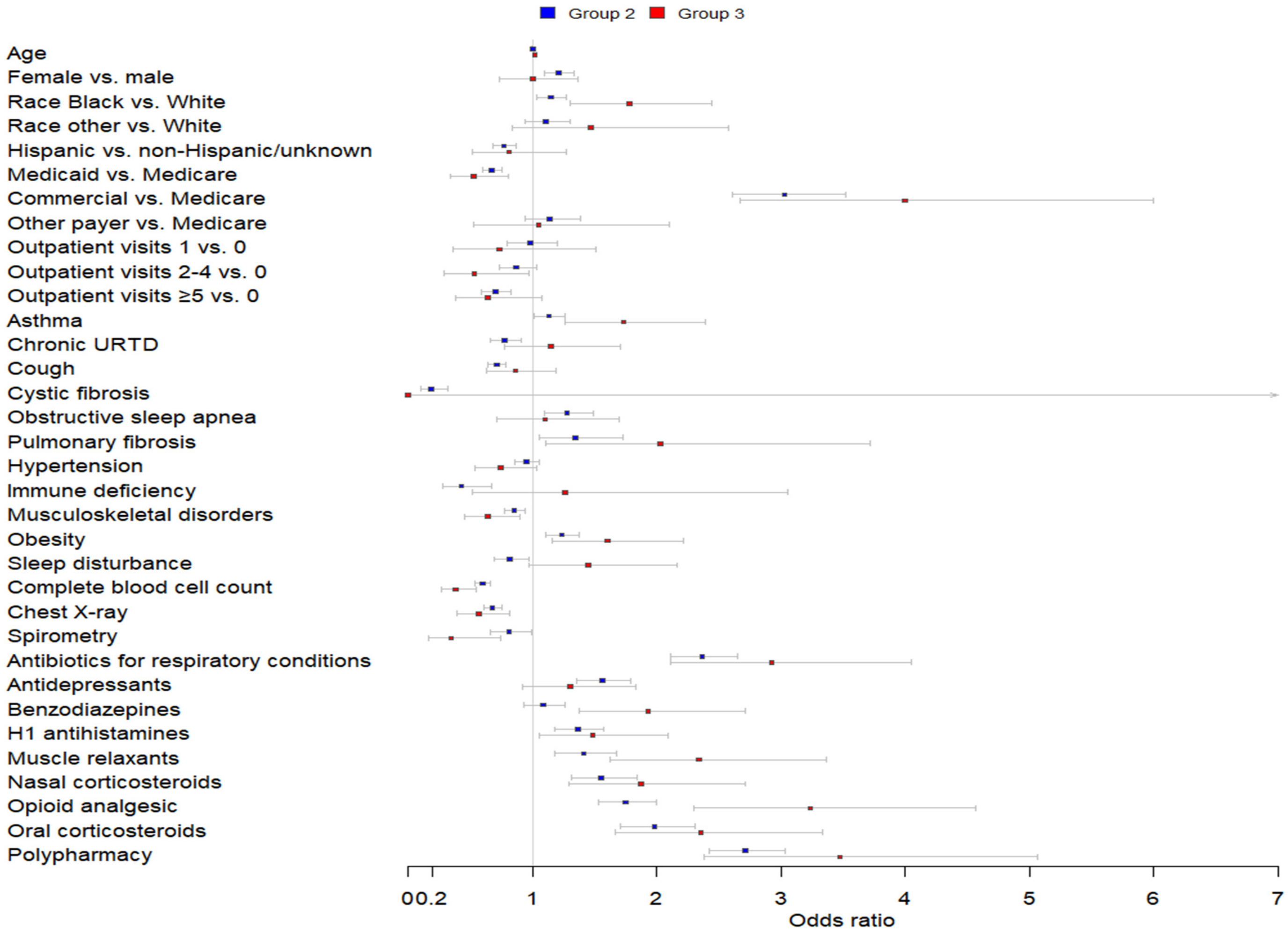

Journal of
Clinical Medicine

Figure S10. Trajectories of opioid antitussive utilization: 2012–2021 OneFlorida data

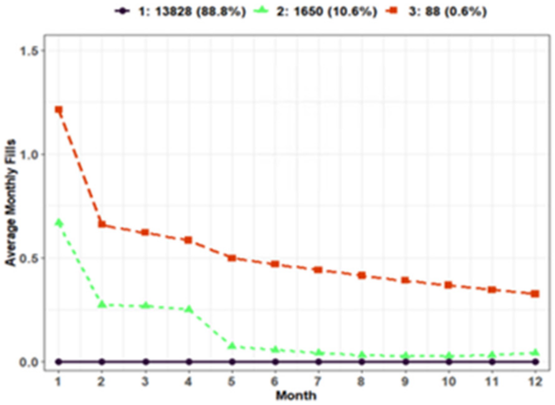

(a) In CC patients

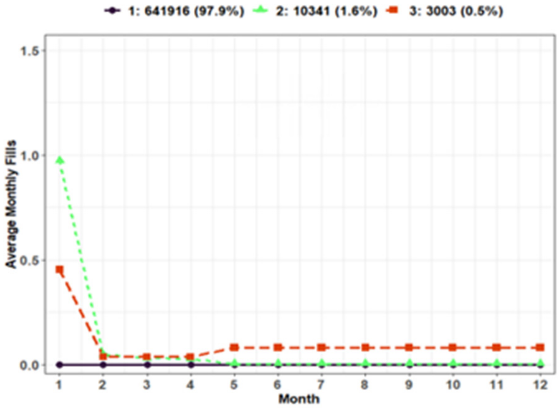

(b) In non-CC patients with any cough-related diagnosis

Abbreviations: CC: Chronic Cough. (a) Three distinct trajectories were identified in CC patients; (b) Three distinct trajectories were identified in non-CC patients with any cough-related diagnosis.

Journal of
Clinical Medicine

Figure S11. Trajectories of benzonatate utilization: 2012–2021 OneFlorida data

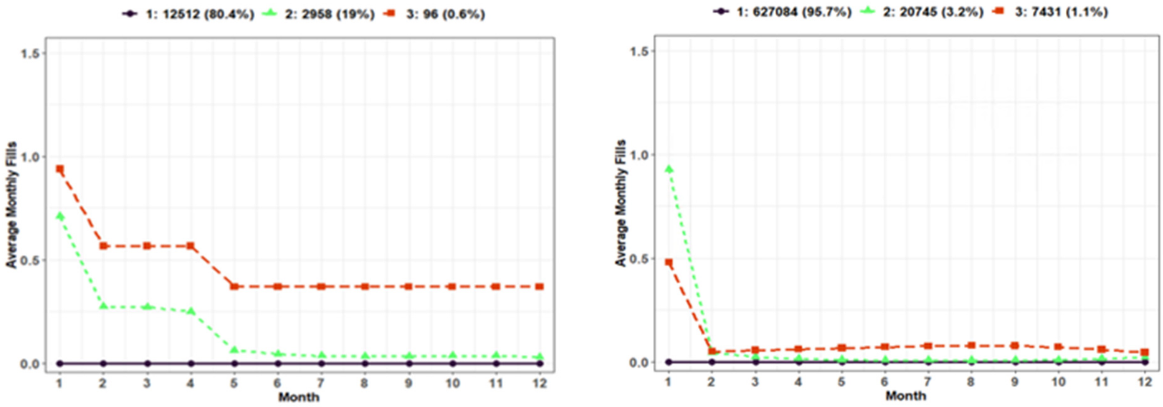

(a) In CC patients

(b) In non-CC patients with any cough-related diagnosis

Abbreviations: CC: Chronic Cough. (a) Three distinct trajectories were identified in CC patients; (b) Three distinct trajectories were identified in non-CC patients with any cough-related diagnosis.

Journal of
Clinical Medicine

Figure S12. Trajectories of dextromethorphan-containing medication utilization

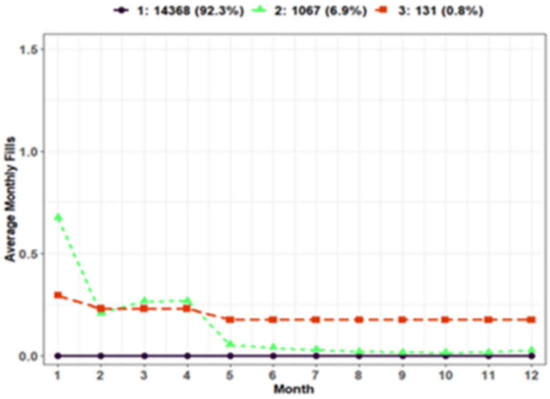

(a) In CC patients

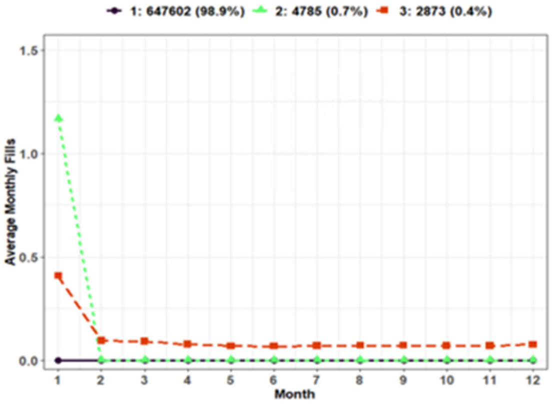

(b) In non-CC patients with any cough-related diagnosis

Abbreviations: CC: Chronic Cough. (a) Three distinct trajectories were identified in CC patients; (b) Three distinct trajectories were identified in non-CC patients with any cough-related diagnosis.

Journal of
Clinical Medicine

MDPI

Figure S13. Trajectories of gabapentinoid utilization

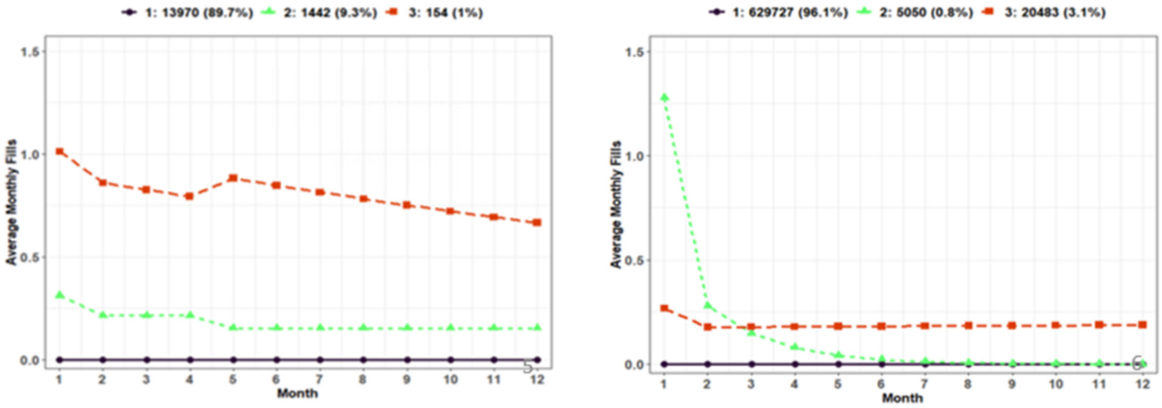

(a) In CC patients

(b) In non-CC patients with any cough-related diagnosis

Abbreviations: CC: Chronic Cough. (a) Three distinct trajectories were identified in CC patients; (b) Three distinct trajectories were identified in non-CC patients with any cough-related diagnosis.

Journal of
Clinical Medicine

Figure S14. Trajectories of cough medication (CM) utilization including gabapetinoids

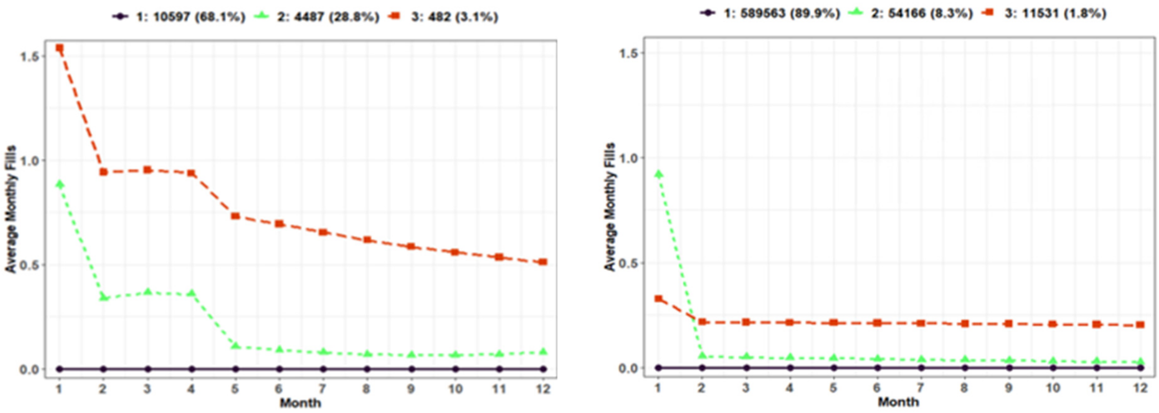

(a) In CC patients

(b) In non-CC patients with any cough-related diagnosis

Abbreviations: CC: Chronic Cough. (a) Three distinct trajectories were identified in CC patients; (b) Three distinct trajectories were identified in non-CC patients with any cough-related diagnosis.
